# Exploring the impact of tertiary lymphoid structures maturity in NSCLC: insights from TLS scoring

**DOI:** 10.3389/fimmu.2024.1422206

**Published:** 2024-09-20

**Authors:** Julie Berthe, Pawan Poudel, Felix J. Segerer, Emily C. Jennings, Felicia Ng, Michael Surace, Alma Andoni, Marco Testori, Megha Saraiya, Miljenka Vuko, Harald Hessel, Mari Heininen-Brown, Jorge Blando, Emma V. Jones, Sophie E. Willis, Jérôme Galon, Rieneke van de Ven, Tanja D. de Gruijl, Helen K. Angell

**Affiliations:** ^1^ Translational Medicine, Oncology R&D, AstraZeneca, Cambridge, United Kingdom; ^2^ Oncology Data Science, Oncology R&D, AstraZeneca, Cambridge, United Kingdom; ^3^ Computational Pathology, Oncology R&D, AstraZeneca, Munich, Germany; ^4^ Oncology Data Science, Oncology R&D, AstraZeneca, Waltham, MA, United States; ^5^ Translational Medicine, Oncology R&D, AstraZeneca, Gaithersburg, MD, United States; ^6^ INSERM, Laboratory of Integrative Cancer Immunology, Paris, France; ^7^ Sorbonne Université, Université Paris Cité, Centre de Recherche des Cordeliers, Paris, France; ^8^ Equipe Labellisée Ligue Contre le Cancer, Paris, France; ^9^ Department of Otolaryngology, Head and Neck Surgery, Amsterdam UMC, Vrije Universiteit Amsterdam, Amsterdam, Netherlands; ^10^ Cancer Center Amsterdam, Cancer Biology and Immunology Theme, Amsterdam, Netherlands; ^11^ Amsterdam Institute for Immunology and Infectious Diseases, Amsterdam, Netherlands; ^12^ Department of Medical Oncology, Amsterdam UMC, Vrije Universiteit Amsterdam, Amsterdam, Netherlands

**Keywords:** NSCLC, tertiary lymphoid structures, tissue scoring, tumor immunity, multiplex immunofluorescence

## Abstract

Tertiary Lymphoid Structures (TLS) are lymphoid structures commonly associated with improved survival of cancer patients and response to immunotherapies. However, conflicting reports underscore the need to consider TLS heterogeneity and multiple features such as TLS size, composition, and maturation status, when assessing their functional impact. With the aim of gaining insights into TLS biology and evaluating the prognostic impact of TLS maturity in Non-Small Cell Lung Carcinoma (NSCLC), we developed a multiplex immunofluorescent (mIF) panel including T cell (CD3, CD8), B cell (CD20), Follicular Dendritic cell (FDC) (CD21, CD23) and mature dendritic cell (DC-LAMP) markers. We deployed this panel across a cohort of primary tumor resections from NSCLC patients (N=406) and established a mIF image analysis workstream to specifically detect TLS structures and evaluate the density of each cell phenotype. We assessed the prognostic significance of TLS size, number, and composition, to develop a TLS scoring system representative of TLS biology within a tumor. TLS relative area, (total TLS area divided by the total tumor area), was the most prognostic TLS feature (C-index: 0.54, p = 0.04). CD21 positivity was a marker driving the favorable prognostic impact, where CD21^+^ CD23^-^ B cells (C-index: 0.57, p = 0.04) and CD21^+^ CD23^-^ FDC (C-index: 0.58, p = 0.01) were the only prognostic cell phenotypes in TLS. Combining the three most robust prognostic TLS features: TLS relative area, the density of B cells, and FDC CD21^+^ CD23^-^ we generated a TLS scoring system that demonstrated strong prognostic value in NSCLC when considering the effect of age, sex, histology, and smoking status. This TLS Score also demonstrated significant association with Immunoscore, EGFR mutational status and gene expression-based B-cell and TLS signature scores. It was not correlated with PD-L1 status in tumor cells or immune cells. In conclusion, we generated a prognostic TLS Score representative of the TLS heterogeneity and maturity undergoing within NSCLC tissues. This score could be used as a tool to explore how TLS presence and maturity impact the organization of the tumor microenvironment and support the discovery of spatial biomarker surrogates of TLS maturity, that could be used in the clinic.

## Introduction

Several spatial biomarkers, predictive of improved patient survival and response to immuno-therapies, have been identified over the last decade. One example is the density and location of immune cells within the tumor microenvironment. In particular, the presence of tumor-infiltrating lymphocytes (TILs) has been correlated to a better prognosis and response to immunotherapy in various cancer types, including melanoma, non-small cell lung cancer, and bladder cancer (1, 2). Other studies have demonstrated similar prognostic and predictive impact of immune cell density within the tumor center or the invasive margin (2, 3). Additionally, the spatial organization of immune cells within the tumor microenvironment can be of major importance. For example, the presence of highly organized ectopic lymphoid structures, called tertiary lymphoid structures (TLS) found in inflamed or tumor tissues, have been linked with better prognosis and response to immunotherapy in many cancer types (4–9).

While TLS are associated with favorable outcomes in tumor indications such as lung, colorectal, and pancreatic cancers, their relationship with histopathological and clinical parameters is complex and can vary across tumor types. In other tumor indications, TLS impact can be controversial, for instance in invasive breast cancer, bladder cancer, and gastric tumors where the presence of TLS has been correlated with poor prognostic value (8, 9). These conflicting observations suggest that not all lymphoid aggregates are functionally equivalent, and minimal characteristics may be required to define a functional TLS. Many cell types are recruited and segregated in two distinct B cell and T cell areas that typically comprise a TLS, including immune cells such as B cells, T cells, mature dendritic cells (mDC), Follicular helper T cells (TFH) and macrophages, differentiated stromal components (follicular dendritic cells (FDC), fibroblastic reticular cells (FRC), marginal reticular cells (MRC)), and high endothelial venules (HEV) (8). This complex TLS structural organization is critical for immune activation, as it enables the interaction of immune cells and antigens, leading to the generation of effective anti-tumor immune responses (8). The presence or absence of some cellular components may reflect different levels of organization, maturation stages or even types of lymphoid aggregates (10–13) which may impact their clinical significance.

Furthermore, it is of importance to note that there is no single, universally accepted definition of TLS, and different groups may define them differently based on their research question or tissue type. Moreover, various methods are used to evaluate and classify TLS, including histology H&E staining, immunohistochemistry (IHC), multiplex immunofluorescence (multiplex IF) and gene expression profiling, each method having its strengths and limitations (8, 9). Using different methods or single IHC markers to identify TLS may lead to variations in the classification of TLS and in the assessment of their clinical impact, with each method focusing on a specific TLS feature.

Variable numbers of lymphoid aggregates can be present within a tumor tissue, each one of these structures displaying unique characteristics (such as the size, cellular composition, location, maturation stage), and each feature having a potential impact on the clinical outcome. A better understanding of TLS impact on patient survival would thus require characterizing them at a high-resolution level by considering their functional, compositional, and spatial characteristics.

The objective of this study was to assess the prognostic value of TLS in Non-Small Cell Lung Cancer (NSCLC), by establishing a TLS Score that reflected the diversity of lymphoid clusters within a tissue. This scoring method considers various factors, including the size of the TLS relative to the tumor size, their cellular composition, and their prevalence.

## Materials and methods

### Acquisition of samples

All human tissues were obtained with fully informed consent and transferred to AstraZeneca. AstraZeneca has a governance framework and processes to ensure that commercial sources have appropriate patient consent and ethical approval in accordance with the principles outlined in the Declaration of Helsinki, in place for collection of the samples for research purposes including use by for-profit companies. The AstraZeneca Biobank in the UK is licensed by the Human Tissue Authority (License No. 12109) and has National Research Ethics Service Committee (NREC) approval as a Research Tissue Bank (RTB) (REC No 17/NW/0207) which covers the use of the samples for this project.

### Immunohistochemistry staining

Immunohistochemistry (IHC) staining was used as a Gold Standard to validate the multiplex immunofluorescence staining. IHC was performed on 4 μm thick sections of FFPE tissues and carried out on BOND RX using the following pre-programmed protocols and ready-to-use reagents (Leica Biosystems): dewax, ER1 citrate-based pH 6 retrieval (CD8, CD20, CD21, and CD23) or ER2 EDTA based pH 9 retrieval (CD3 and DC-LAMP) at 100°C for 20 mins. A blocking step using Protein Block Serum-Free reagent (Agilent) preceded the ‘F standard’ staining protocol, with post-primary (CD8, CD20, CD21 stains) or without (CD3, CD23 stains), poly-HRP-IgG and DAB refine (Polymer refine detection kit, Leica Biosystem). Primary antibodies used were as follows: anti-DC-LAMP (clone 1010E1.01, Dendritics, at 1 μg/mL), anti-CD3 (clone 2GV6, Roche, at 0.1 μg/mL), anti-CD8 (clone C8/144 B, Dako, at 1.5 μg/mL), anti-CD20 (clone L26, Abcam, at 0.1 μg/mL), anti-CD21 (clone 2G9, Cell Marque, at 0.5 μg/mL), anti-CD23 (clone SP23, Abcam, at 0.25 μg/mL). The antibodies were diluted in Dako antibody diluent (Agilent) and incubated for 15 mins. For DC-LAMP staining, ER2 retrieval incubation time was 40 mins, primary antibody incubation 40 mins and the secondary antibody used was a donkey anti-rat IgG H&L HRP (Abcam) at 1/200 dilution incubated 16 mins. Digital slide images were acquired with the Aperio AT2 scanner (Leica) using a 20x or 40x objective.

### PD-L1 and immunoscore (CD3 and CD8) immunohistochemistry

Tumor sections were stained by IHC using the VENTANA PD-L1 (SP263) assay and scored by a pathologist for the proportion of membrane staining tumor cells and immune cells as described in Scorer et al. (14). CD3 and CD8 IHC Immunoscore Gold Standard assays were performed using the VENTANA, in conformity with Pages et al. (15). The T cells CD8^+^ density in the tumor center and the invasive margin is used to provide an Immunoscore ‘I’. It ranges from Immunoscore 0 ‘I0’ to Immunoscore 4 ‘I4’ depending on the T cell density in both tumor regions.

### Multiplex immunofluorescence staining and multispectral image acquisition

Multiplex IF staining was conducted on 4 μm thick sections from FFPE NSCLC tissues using the Opal 6-Plex Detection Kit for Whole Slide Imaging (Akoya Biosciences). The BOND RX automated stainer was used for the pretreatment and staining of the tissues using ER2 retrieval at 100°C for 40 mins (Leica Biosystems). The endogenous peroxidase was blocked using the Peroxidase Block Novocastra (Leica) for 7 mins, before staining the tissues through repeated staining cycles for each marker. Each staining step cycle was composed of 5 steps: protein blocking using the Antibody Diluent/Block reagent for 5 mins (Akoya Biosciences), primary antibody incubation for 45 mins to 60 mins, secondary antibody incubation for 10 mins, Opal dye incubation for 10 mins, and an antibody denaturation step using ER1 retrieval at 97°C for 20 min to 30 mins. Identical primary antibody clones and concentrations were used for both chromogenic IHC and multiplex IF staining. The staining order and antibody-TSA reagent combination were as follows: 1) anti-DC-LAMP visualized with Opal480 (1/200 dilution), 2) anti-CD3 with Opal690 (1/100 dilution), 3) anti-CD8 with Opal520 (1/150 dilution), 4) anti-CD20 with Opal570 (1/100 dilution), 5) anti-CD21 with Opal620 (1/100 dilution), and 6) anti-CD23 with TSA-DIG (1/100 dilution) and Opal780 (1/50 dilution). Every TSA reagent was double dispensed. The Opal polymer anti-Ms + Rb HRP secondary antibody (Akoya Biosciences) was used for CD3, CD20, CD21 and CD23 stainings, the anti-mouse HRP SignalStain Boost IHC Detection Reagent (Cell Signaling Technologies) for CD8 staining, and the donkey anti-rat IgG H&L HRP (Abcam) for DC-LAMP staining. At the end of the protocol, the stained slides were counterstained with DAPI. The slides were scanned at 20x using the PhenoImager automated imaging system (Phenoptics; Akoya Biosciences) and multispectral images were unmixed using the InForm software version 2.4.8 and the synthetic spectral library (Akoya Biosciences).

### Image analysis and TLS/B-cell cluster detection

A robust, standardized, and scalable image analysis pipeline has been developed and applied to analyze the 406 resection images stained with our panel across the entire tissue area (**Supplementary Figure 1**). To account for variability in staining intensities across staining batches we initially normalized intensities utilizing batch controls from the same tissue block (**Supplementary Figure 1A**). We adjusted equalization settings for each of the control slides such that the individual markers in the control block appear at the same intensity across all staining batches. Afterwards, the equalization settings of each control slide were applied to all slides of the corresponding staining batch. This was followed by image analysis (**Supplementary Figure 1B**), where, in an initial step, annotations of the tumor center “TC” and invasive margin “IM” were manually drawn by a pathologist for each image. These annotations served as regions of interest (ROIs) for further analysis. The denominated Annotated Area “AA” corresponds to the entire tumor tissue which includes IM and TC areas. Afterwards, we applied several deep-learning-based segmentation and detection models across the whole slides for purposes of: (i) detection of nuclei, (ii) detection of positive cells for all markers in the panel, (iii) segmentation of analyzable tissue, and (iv) segmentation of parenchymal regions based on synthetically generated PANCK staining (16). Afterwards, TLS were segmented through the detection of CD20^+^ cell clusters and enlarging these regions into the surrounding areas of high CD3^+^ densities to detect both the TLS B-cell (CD20^+^) and T-cell (CD3^+^) zones (**Supplementary Figure 1C**). Clusters containing less than 20 CD20^+^ cells arbitrarily were not considered as TLS. Image analysis results for regions and detected cells were then combined into a single segmentation map and cells were classified such that information about marker positivity and region associations were available for each detected cell. Finally, readouts were calculated across different ROIs, as summarized in **Table 1**.

**Table 1:** Image analysis readouts.

Tissue detection		Tissue areas detected: 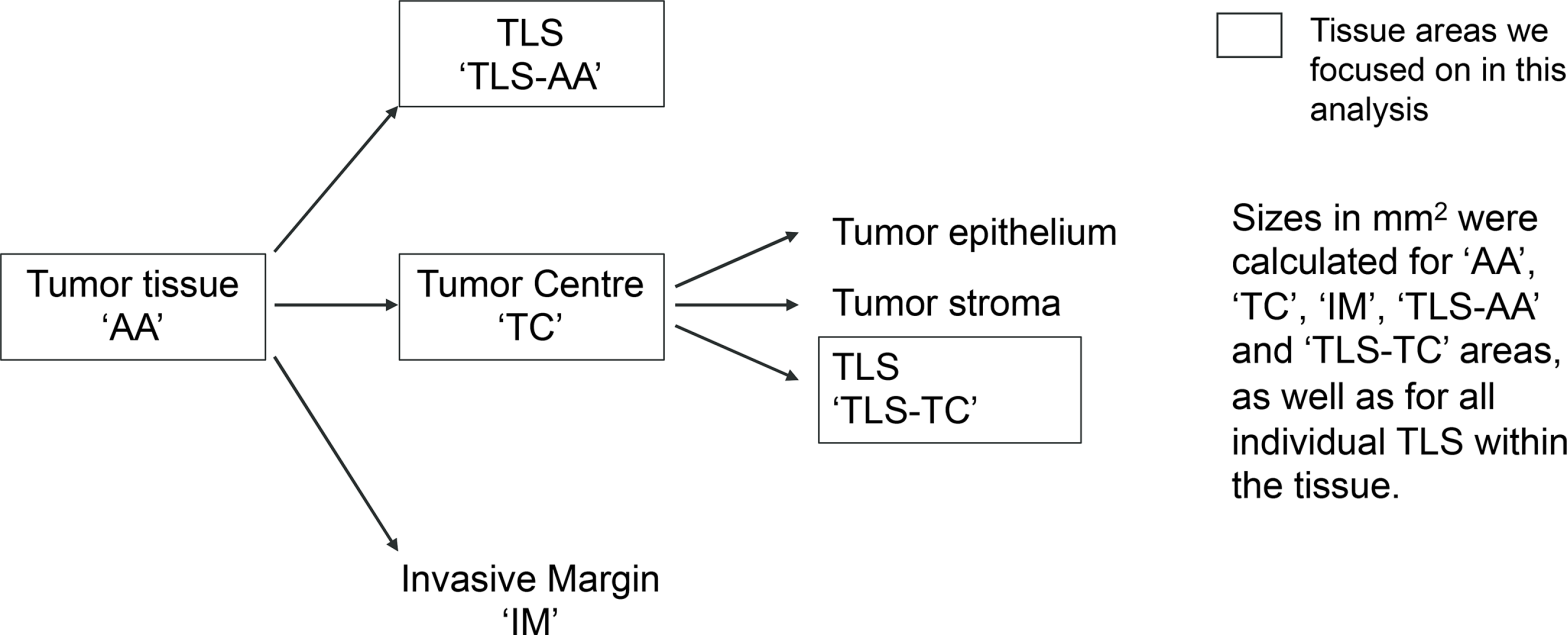	
B-cell cluster / TLS composition and maturation status	B-cell zone	**B-cell zone and Germinal centre staining: CD20, CD21 and CD23 markers**	
		CD20: pan B-cell marker CD21 and CD23: B cells and Follicular Dendritic Cells (FDC) (8)	
		TLS maturation stage according to CD21 and CD23 positivity status: (10) TLS CD21^-^ CD23^-^: B-cell cluster TLS CD21^+^ CD23^-^: Primary follicle-like TLS (‘immature TLS’) TLS CD21^+^ CD23^+^: Secondary follicle-like TLS (‘mature TLS’)	
		**B cell phenotyping**	**B cell populations**
		CD20^+^ CD21^-^ CD23^-^	B cells CD21^-^ CD23^-^
		CD20^+^ CD21^+^ CD23^-^	B cells CD21^+^ CD23^-^
		CD20^+^ CD21^-^ CD23^+^	B cells CD21^-^ CD23^+^
		CD20^+^ CD21^+^ CD23^+^	B cells CD21^+^ CD23^+^
		**FDC phenotyping**	**FDC populations**
		CD3^-^ CD20^-^ CD21^+^ CD23^-^	FDC CD21^+^ CD23^-^
		CD3^-^ CD20^-^ CD21^-^ CD23^+^	FDC CD21^-^ CD23^+^
		CD3^-^ CD20^-^ CD21^+^ CD23^+^	FDC CD21^+^ CD23^+^
	T-cell zone	**T-cell zone staining: CD3, CD8 and DC-LAMP markers**	
		**T cell phenotyping**	**T cell populations**
		CD8^+^	Cytotoxic CD8^+^ T cells
		CD3^+^ CD8^-^	Surrogate for CD4^+^ T cells
		**Dendritic cell phenotyping**	**DC population**
		DC-LAMP^+^	mature dendritic cells (mDC) DC-LAMP^+^
			* readouts within TLS area only (to limit the challenge of the expression in both mDC and epithelial pneumocytes II cells)
	The density of each cell population was analysed per tissue area and per TLS.		

### Heatmap visualization and principal component analysis of multiplex IF density readouts

The cell density of each cell phenotype (N=10) within the regions labelled ‘AA’ (entire tumor annotated area), ‘AA-TC’ (tumor center area), ‘TLS-AA’ (TLS within the ‘AA’ area) and ‘TLS-TC’ (TLS within the ‘TC’ area) were log_10_ transformed and scaled to normalize the data. ‘TLS-AA’ and ‘TLS-TC’ correspond to TLS specific areas detected by the image analysis solution. Hence, when no TLS were detected within the tissue (TLS negative cases), cell densities were treated as missing values (*NA*) in those TLS specific areas. Principal component analysis was performed using the R package – *FactoMineR* (17). Spearman’s correlation was used to assess the correlation between TLS readouts, including densities of immune phenotypes and TLS features, with the first five principal components.

### Immune gene expression profiling and gene signature calculation

5–10 µm thick sections from 375 primary FFPE NSCLC tumor tissues were used for NanoString gene expression (GE) analysis. Tumor macro-dissected and RNA extracted with RNeasy FFPE extraction kit (Qiagen). NanoString transcriptomics using both the PanCancer Immune Profiling Panel and Myeloid Innate Immunity Panel (770 genes each) was carried out following manufacturer’s instructions. The data obtained were processed using the nSolver Analysis Software version 4.0 (https://www.nanostring.com/products/analysis-software/nsolver) (NanoString). The processed NanoString data were used to estimate the signature scores associated with the B cells and TLS chemokine using the R package GSVA (18).

### *EGFR* mutation status

*EGFR* mutation status was determined as described in Tu et al. (19). Briefly, *EGFR* mutant tumors were identified by annotation from the tissue vendors or verified internally by whole exome sequencing.

### Statistical analysis

All the statistical analyses in this study were performed using R software (version 4.1.0). Differences between groups (or clusters) were tested with the Wilcoxon rank sum test and the Kruskal–Wallis test for comparisons between two or more groups, respectively. To identify the optimal cut-off points for converting the continuous TLS score into categorical scores, an iterative log-rank test was performed using the Python package *lifelines* (20). All other survival analyses were done using the R package – *survival* (21). Survival plots were generated using the R package – *survminer* (22)*. Concordance index was estimated using R package – dynpred* (23).

## Results

### Multiplex IF panel deployment across 408 primary NSCLC tissue resections

**Figure 1:**
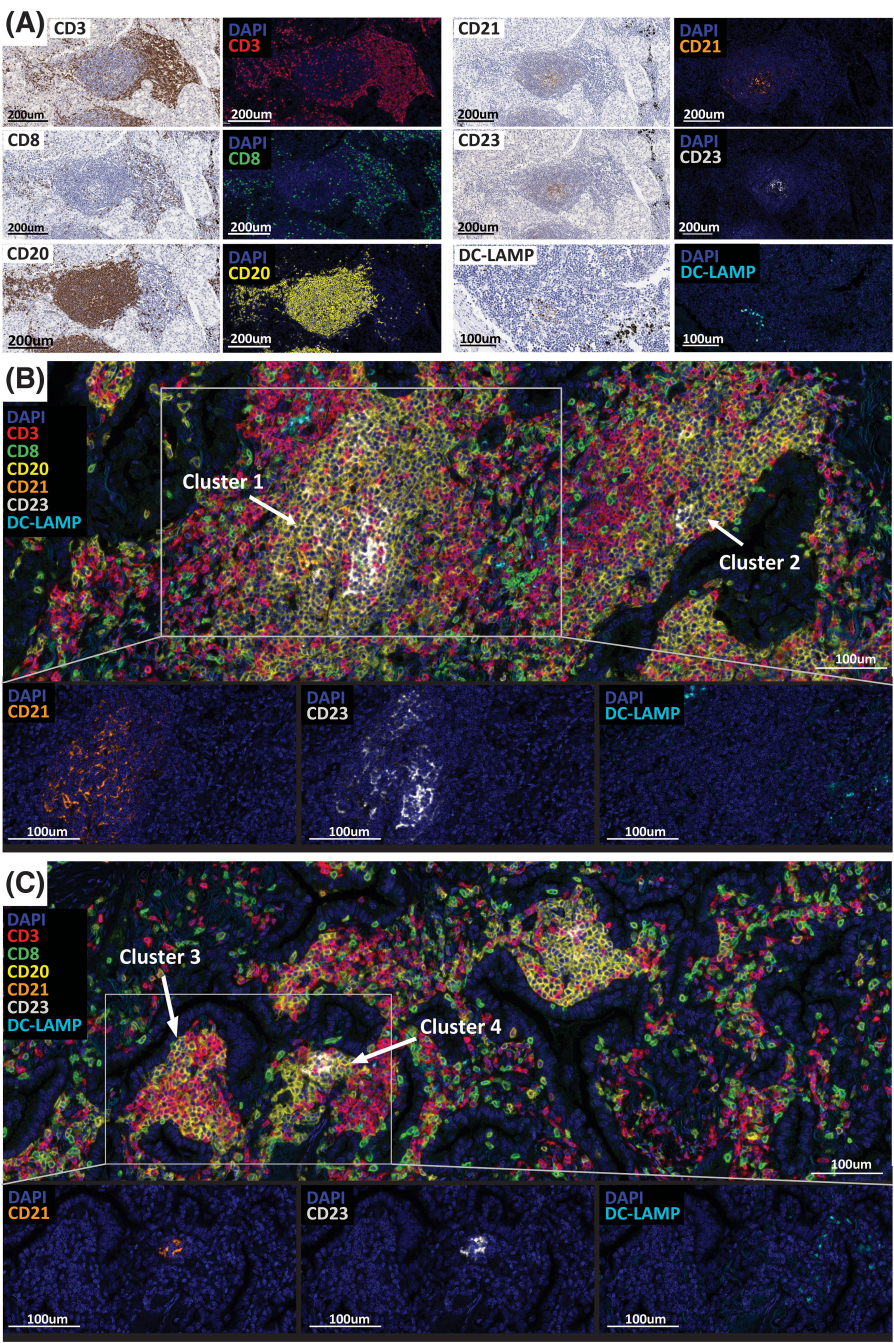
TLS detection and maturity assessment in 406 NSCLC cases. **(A)** Multiplex immunofluorescence assay validation (CD3, CD8, CD20, CD21, CD23, DC-LAMP) by comparing the IF to the chromogenic IHC staining performed on serial tissue sections. CD3 (T cells, red), CD8 (CTL T cells, green), CD20 (B cells, yellow), CD21 (FDC, orange), CD23 (GC B cells, white), DC-LAMP (mDC, cyan). **(B, C)** Representative images of large **(B)** and small **(C)** TLS/B-cell clusters positive for the maturation markers CD21, CD23 and DC-LAMP detected in NSCLC cases. Slides were imaged using the PhenoImager HT automated imaging system. Scale bars are indicated.

In order to work towards a more standardized characterization of TLS, we here assessed the clinical impact of TLS parameters such as their size, cellular composition, and maturity status, understand the added value of each parameter and highlight any overlap in the information carried by these parameters. To this end, we developed a multiplex IF panel that enabled simultaneous detection of the TLS main cellular components: CD20 for B cells (TLS B-cell zone), CD3 for T cells and CD8 for cytotoxic T cells (TLS T-cell zone), and maturation markers such as DC-LAMP for activated conventional dendritic cells, CD21 for follicular dendritic cells (FDC) and CD23 for mature B cells. It is important to note that CD21 and CD23 markers can be expressed on both FDC and B cell populations (8, 24–26) (**Table 1**). The multiplex IF protocol was validated by comparing the IF staining against the single IHC chromogenic staining which was considered as the Gold Standard reference, performed on consecutive tissue sections (**Figure 1A**). The panel was then deployed across a cohort of 408 primary NSCLC baseline tumor resections, from patients treated with standard of care agents. Two cases were excluded from the analysis due to staining quality issues. The cohort clinicopathological characteristics are detailed in **Table 2**.

**Table 2:** Clinical and histologic characteristics of the NSCLC patient cohort (N = 406).

Clinical characteristics		Prognostic variables	
Characteristic	N = 406	Characteristic	N = 406
**Diagnosis category*^1^***		**Survival status*^1^***	
adenocarcinoma	214 (53%)	Alive	171 (42%)
adenosquamous	1 (0.2%)	Deceased	188 (46%)
bronchioalveolar	1 (0.2%)	Unknown	47 (12%)
large cell	6 (1.5%)	**Overal survival (months)*^2^***	23 (9, 45)
other	9 (2.2%)	Unknown	48
sarcomatoid	1 (0.2%)	**Progression free survival*^2^***	19 (8, 40)
squamous cell	142 (35%)	Unknown	66
Unknown	32 (7.9%)	**Censoring status (PFS)*^1^***	101 (30%)
**Tumor grade*^1^***		Unknown	67
G1	9 (2.2%)	**Recurrence*^1^***	
G1-2	8 (2.0%)	Never disease-free	4 (1.0%)
G2	69 (17%)	No	239 (59%)
G2-3	10 (2.5%)	Unknown	57 (14%)
G3	83 (20%)	Yes	106 (26%)
Unknown	227 (56%)	*^1^* n (%); *^2^* Median (IQR)	
**T Category*^1^***			
T1	106 (26%)		
T2	191 (47%)	**Patient demographic and data information**	
T3	59 (15%)	**Characteristic**	**N = 406**
T4	10 (2.5%)	**Age*^2^***	67 (61, 74)
TA	1 (0.2%)	Unknown	32
Unknown	39 (9.6%)	**Sex*^1^***	
**M category*^1^***		Female	166 (41%)
M0	59 (15%)	Male	208 (51%)
M1	6 (1.5%)	Unknown	32 (7.9%)
Unknown	341 (84%)	**Race*^1^***	
**N category*^1^***		Asian	11 (2.7%)
N0	234 (58%)	Caucasian	49 (12%)
N1	69 (17%)	Unknown	346 (85%)
N2	46 (11%)	**Smoking history*^1^***	
Unknown	57 (14%)	Current	110 (27%)
**Stage*^1^***		Never	38 (9.4%)
I	83 (20%)	Past	175 (43%)
II	58 (14%)	Unknown	83 (20%)
III	17 (4.2%)	**Alcohol history*^1^***	
IV	3 (0.7%)	Heavy Drinker	2 (0.5%)
Unknown	245 (60%)	Never	18 (4.4%)
**Chemo treatment history*^1^***		Social or Occasional Drinker	21 (5.2%)
No	48 (12%)	Unknown	365 (90%)
Unknown	249 (61%)	**Exome Profiling^1^**	273 (67%)
Yes	109 (27%)	**Nanostring Profiling*^1^***	375 (92%)
**Radiation treatment history*^1^***		*^1^* n (%); *^2^* Median (IQR)	
No	46 (11%)		
Unknown	309 (76%)		
Yes	51 (13%)		
**TLS status*^1^***			
TLS Negative	134 (33%)		
TLS Positive	272 (67%)		
**TLS count*^2^***	2 (0, 8)		

*^1^* n (%); *^2^* Median (IQR).

A visual inspection conducted by pathologists revealed a large variety of B-cell clusters/TLS located within the tumor tissue exhibiting different sizes, organizational patterns, and cellular composition (**Figures 1B, C**). Despite diameter sizes varying from approximatively 150-200 mm to > 500 mm, all clusters presented a clear CD20^+^ B-cell zone surrounded by a CD3^+^ T-cell zone containing CD3^+^ CD8^+^ cytotoxic T cells and a high density of CD3^+^ CD8^-^ cells considered as CD4^+^ T cells (**Figure 1B**). We found the degree of maturity of an aggregate, assessed by the presence of positive cells for CD21 and CD23 markers, to be independent of their size. Example images of both large (> 500 mm) and small (150-200 mm) B-cell clusters are presented in **Figures 1B, C**, respectively. **Figure 1B** shows two large clusters, (Cluster #1 and Cluster #2), describing different levels of maturation. Cluster #1 presented a central area with high prevalence of CD21^+^ and CD23^+^ cells, corresponding to the follicular dendritic cells network and the germinal center, also known as site of an ongoing local B cell activation process that leads to the differentiation of specific memory B cells and plasma cells. In contrast, while located in the same tumor area, Cluster #2 shows a very low prevalence of cells positive for those maturity markers. Both aggregates are positive for DC-LAMP, suggesting the presence of mature dendritic cells. However, it is important to note that the image analysis of DC-LAMP in NSCLC was challenged by the presence of pneumocytes type II epithelial cells that could also express this marker. Thus, we cannot be certain that the DC-LAMP^+^ cells detected are all activated dendritic cells, and the results should be interpreted carefully. Similar to large clusters, small structures (**Figure 1C**) show different maturation levels. Indeed, whilst Cluster #3 does not contain any positive cells for CD21, CD23 and DC-LAMP, Cluster #4 presents high densities of CD21^+^, CD23^+^ and DC-LAMP^+^ cells. These observations suggest that a high organizational and maturation degree of B-cell clusters, with the presence of a follicular dendritic cells network and a germinal center, is independent of their size.

Moreover, if each lymphoid aggregate displays unique features that could result in different anti-tumor immune functions and clinical outcomes (8, 9, 27), it is worth noting that a tumor tissue can contain multiple aggregates, all describing specific size, cellular composition, organization and maturation levels (**Figures 1B, C**). Thus, when assessing the clinical value of B-cell aggregates, it is important to consider all types of aggregates present within a tissue.

Following this visual assessment identifying small B-cell aggregates positive for CD21, CD23 and DC-LAMP, and with the objective of evaluating how the size, cellular composition and maturation degree impact the immune cell activity and clinical outcomes, we developed an image analysis solution that specifically detects B-cell aggregates containing at least 20 CD20^+^ cells (**Supplementary Figure 1C**). These aggregates were segmented by detecting clusters of high density CD20^+^ cells and enlarging these regions into the surrounding areas of high CD3^+^ densities. The specificity of this algorithm was confirmed after pathologist visual assessment. Furthermore, in the absence of a universal and specific definition for TLS, we designated ‘TLS’ as large lymphoid aggregates exhibiting a germinal center and displaying CD21, CD23 and DC-LAMP positive cells. The remaining aggregates detected were referred to as ‘B cell aggregates’ or ‘B cell clusters’.

In addition to TLS area, the segmentation of tumor epithelium and the cell phenotyping analysis required the development and deployment of several additional deep-learning-based segmentation and detection models (**Supplementary Figure 1B**). Information about marker positivity and region association were available for each detected cell, hence allowing us to evaluate the impact of TLS composition in an accurate way. Using these data, we calculated densities of each cell type and sub-type within the segmented regions described in **Table 1**. Owing to the considerable dynamic ranges observed in the densities of all cell types within both the total tumor area and the TLS regions, log10 transformation was applied to these densities for subsequent analysis. This transformation was undertaken to normalize the data and stabilize the variance, as illustrated in **Supplementary Figure 2A**.

### NSCLC cohort immune profiling

**Figure 2:**
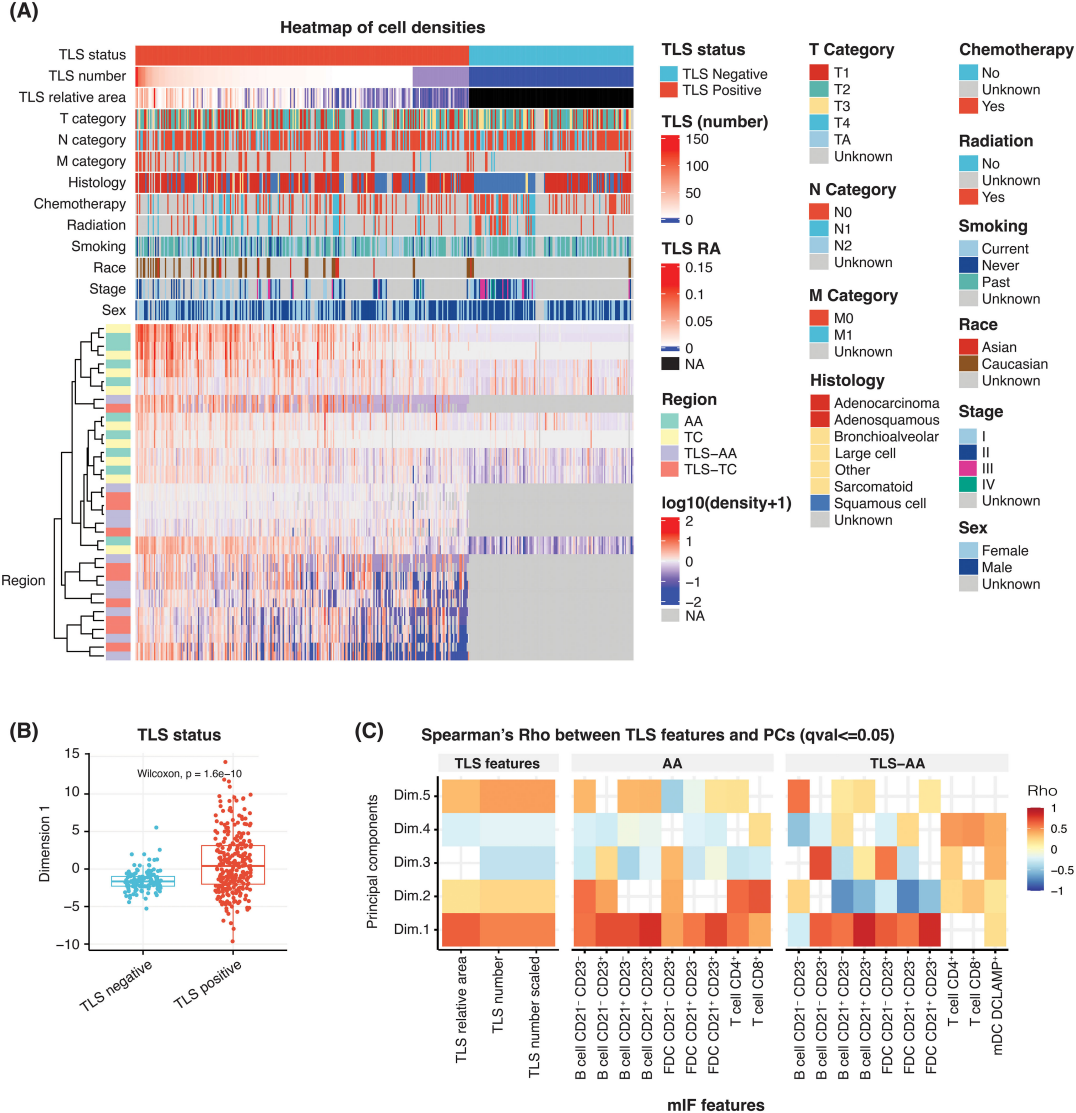
Heatmap of multiplex IF features to identify the key cell phenotypes associated with TLS structures. **(A)** Heatmap showing the scaled (following the log_10_ transformation) density of 10 cell phenotypes and their association with sex, stage, race, smoking status, therapy (radiation and chemotherapy), histology, TNM categories, TLS relative area (RA), TLS number and TLS status. Individual patients are represented in each column. Each row represents the cell density of a specific cell phenotype located within the indicated tissue region. For cases TLS negative, TLS RA and cell densities are treated as missing value *NA*. **(B)** A box plot comparing the first principal component scores according to the TLS status of the NSCLC cases. **(C)** A heatmap showing the correlation between multiple multiplex IF features, namely: TLS relative area, TLS number (unscaled), TLS number (scaled), densities of 10 cell phenotypes in ‘AA’ and ‘TLS-AA’ (*x-axis*), with the first principal component (*y-axis*).

In our effort to understand the level of B-cell cluster and TLS heterogeneity within the tumor TME of NSCLC patients, we generated a heatmap using the TLS multiplex IF readouts (**Figure 2A**). These readouts, summarized in **Table 1**, consisted of the densities of 10 cell phenotypes in different tissue areas and described as the main cellular components of the TLS B-cell zone (B cells and follicular dendritic cells) and the TLS T-cell zone (T cells and mature dendritic cells).

B cells and FDC cell types were segregated into different subsets based on the positivity of specific markers CD20, CD21 and CD23, commonly used to evaluate TLS impact and maturation status in the clinic (5, 7–10, 28, 29). The CD20 antigen is expressed on the surface of B-cells starting from the pre-B cell stage, with the exception of plasmablasts and plasma cells. In contrast, the proteins CD21 and CD23 are expressed at later stages in the activation process. CD21 positivity can be observed from the transitional B cell stage 1, and CD23 expression is typically detected from the transitional B cell stage 2. These markers are commonly used to identify germinal center B cells (25, 30, 31). In this context, B cells were segregated into four B cell subsets depending on CD21 and CD23 positivity status. The subsets are the following: B cells CD21^-^ CD23^-^, B cells CD21^+^ CD23^-^, B cells CD21^-^ CD23^+^ and B cells CD21^+^ CD23^+^. Similarly, we categorized the follicular dendritic cells, which are stromal cells involved in the structure and organization of TLS and which can express both CD21 and CD23, into three subsets based on their positivity status for both CD21 and CD23. Three subtypes were assessed: FDC CD21^+^ CD23^-^, FDC CD21^-^ CD23^+^ and FDC CD21^+^ CD23^+^. Furthermore, the T cell population was divided into two cell populations: cytotoxic T cells CD3^+^ CD8^+^ (CTL) and T cells CD3^+^ CD8^-^ considered as a surrogate of T helper cells CD4^+^.

The densities of B cells, FDC and T cells were analysed in different tissue areas including: the tumor annotated area ‘AA’ corresponding to the entire tumor area, the tumor centre ‘TC’, TLS within the tumor annotated area ‘TLS-AA’ and TLS within the tumor centre ‘TLS-TC’. Finally, mature dendritic cells were assessed using the DC-LAMP marker, commonly used to study this cell population. However, lung tissues may contain type II pneumocytes epithelial cells, which exhibit positivity for this antigen. Consequently, we restricted its analysis to TLS areas, specifically ‘TLS-AA’ and ‘TLS-TC’, mature dendritic cells being more likely to be located within TLS (**Table 1**).

The heatmap analysis considered the TLS multiplex IF cell densities readouts within the tissue areas ‘AA’, ‘TC, ‘TLS-AA’ and ‘TLS-TC’. Individual patients were represented in each column (**Figure 2A**). This analysis predominantly separated the cohort according to the presence or absence of TLS within the tissue, which was an expected result as the assay is specific to the main TLS immune cell components. Additionally, TLS positive cases were segregated according to diverse TLS features such as the TLS number, the immune cell densities and the TLS relative area, the latter corresponding to the total TLS area within a tumor tissue divided by the tumor area (**Supplementary Figure 2B**).

We then proceeded with a principal component analysis (PCA) to understand and identify the key TLS variables driving the observed heterogeneity within tumor tissues. Our analysis revealed that the first dimension effectively distinguished NSCLC cases based on their TLS status, whether TLS-positive or TLS-negative (**Figure 2B**). This first dimension was also significantly correlated with TLS features such as the TLS relative area and TLS number both in unscaled and scaled values (**Figure 2C**). Similarly, most cell densities exhibited a significant correlation with this first dimension and displayed Spearman’s Rho values over 0.5 (**Figure 2C**). We particularly focused on the whole tumor area ‘AA’ and TLS-specific regions within the tumor ‘TLS-AA’ to assess the TLS cellular diversity throughout the entire tissue. Moreover, although additional PCA dimensions allowed us to further explain the variance, the common trend observed among multiplex IF cell density readouts in the first component suggested a shared mechanism influencing their presence in TLS regions (**Figure 2C**).

These heatmap and PCA results supported the TLS heterogeneity observed during the visual inspection amongst this NSCLC cohort and confirmed the need of further characterizing the TLS biology – TLS number, TLS relative area, TLS composition – within a tissue to accurately evaluate their impact on clinical outcomes.

### Prognostic value of TLS features and cell densities

Following the heatmap visualization and PCA analysis, we aimed to understand how the prevalence of each TLS main component, such as B cells, T cells, FDC and dendritic cells, TLS maturation status and location within the tumor tissue, can impact the prognostic relevance of these structures.

**Figure 3:**
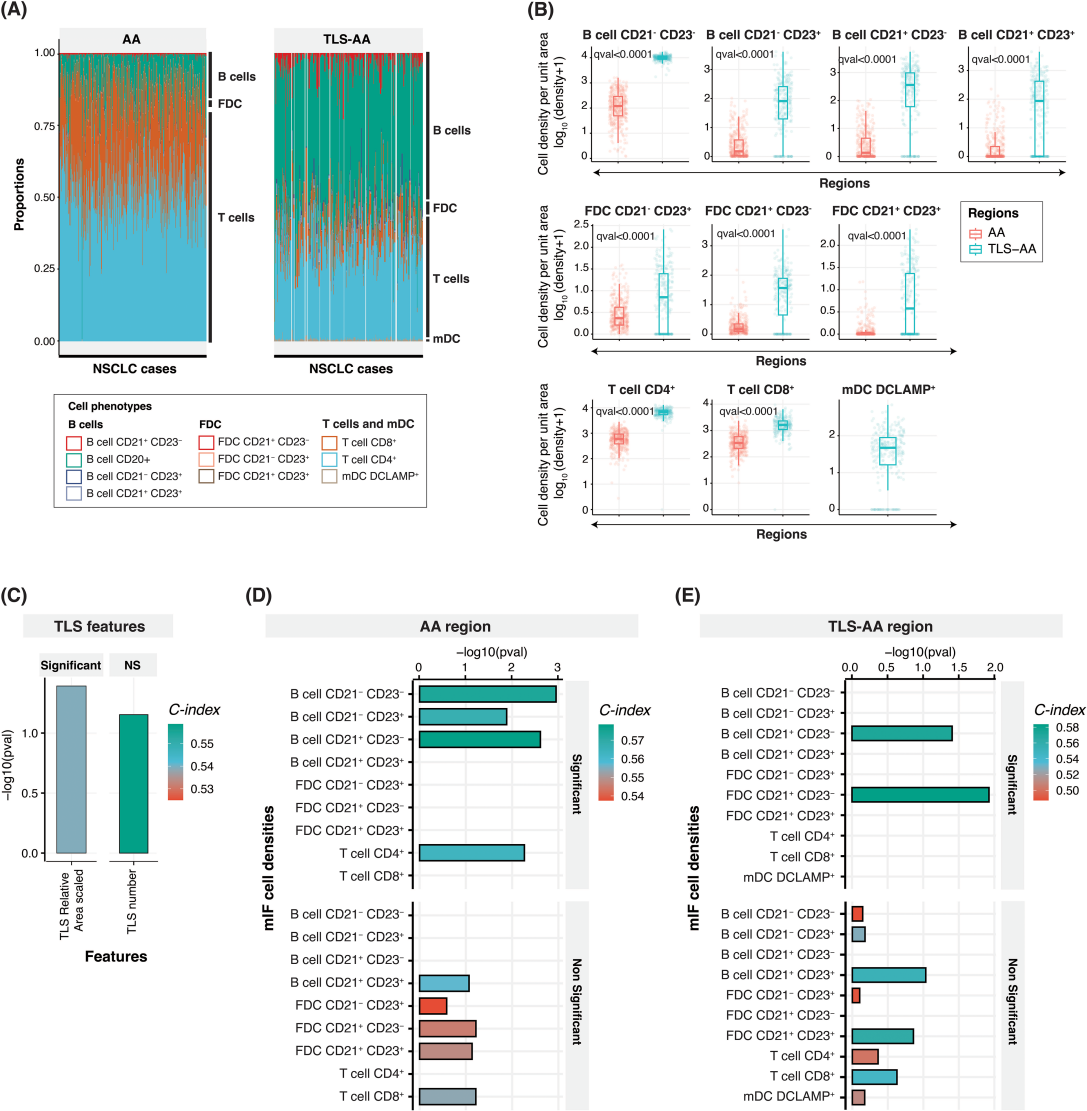
Cell densities within the tumor and TLS-specific area, and prognostic relevance. **(A)** Bar plot showing the relative proportion of ten cell phenotypes captured using the multiplex IF panel in the tumor ‘AA’ (left panel) and TLS-specific area ‘TLS-AA’ (right panel) regions. The proportion has been calculated by dividing the specific cell type density by the total cell density. **(B)** Box plots comparing the densities (number of cells per unit area) of these ten cell phenotypes between the ‘AA’ and ‘TLS-AA’ regions. The q-value in the plot refers to the FDR adjusted p-value from Wilcoxon rank sum test. **(C–E)** Bar plots showing concordance index (C-index), and -log_10_(p-values) for TLS relative area (scaled) and TLS number **(C)** and cell phenotypes within the tumor area ‘AA’ **(D)** or within TLS specific areas ‘TLS-AA’ **(E)**. NS, Non-significant.

The proportion of B cells was significantly increased in the TLS compared to the tumor areas (**Figures 3A, B**). These cells represented approximately 55% and 20% of the total cell population detected within the TLS areas and the tumor, respectively (**Figure 3A**). This result was in coherence with B cells being the main immune component of TLS and our subsequent image analysis strategy to develop a TLS detection algorithm based on the CD20 marker positivity. As shown in **Figure 3B**, all B cell subtypes displayed higher densities in TLS specific areas. Similarly, we evaluated the distribution of different follicular dendritic cell subsets. The density of all FDC subsets was significantly increased in TLS compared to the entire tissue area, the FDC population representing approximately 3% and 6% of the analysed cells within the tumor and TLS specific areas, respectively (**Figures 3A, B**). After evaluating the overall proportion of B cells and FDC, which are the two main cell phenotypes that compose TLS B-cell zone and germinal centre, we assessed the prevalence of the main TLS T-cell zone components, T cells and mature dendritic cells DC-LAMP^+^. We observed a considerably higher proportion of T cells – including both CTL and CD4^+^ cells – in the tumor areas compared to the TLS areas, the T cell population representing approximately 80% of the phenotyped cells within the tumor tissue and 40% in the TLS areas (**Figure 3A**). Interestingly, the density of both CTL and CD4^+^ cell subsets was significantly higher in TLS areas compared to the entire tumor area (**Figure 3B**). Finally, a low prevalence of mature dendritic cells DC-LAMP+ was observed within TLS areas, these cells representing less than 1% of the total TLS cellular population (**Figure 3A**).

We next conducted a comprehensive analysis and calculated the Concordance Index (C-index) of TLS features (**Figure 3C**) and cell densities within two distinct tissue areas, the tumor tissue ‘AA’ (**Figure 3D**) and TLS-specific areas ‘TLS-AA’ (**Figure 3E**), to evaluate their associations with patient survival in our study cohort. The C-index, ranging from 0 to 1, evaluates survival model performance. A score of 1 signifies perfect discrimination between survivors and non-survivors, while 0.5 indicates performance similar to random guessing. The C-index, Hazard ratio, 95% Confidence interval, and p-values of all parameters assessed in multiple tissue areas are also indicated in **Supplementary Table 1**. Surprisingly, we showed that among the two TLS features considered, scaled TLS relative area (scaled) and TLS number, only the TLS relative area demonstrated a significant prognostic power with a C-index of 0.54 (p-value = 0.04) (**Figure 3C** and **Supplementary Table 1**). Moreover, our analysis revealed the importance of considering the location within the tissue when understanding the contribution of specific cell phenotypes to survival outcomes. Among the densities (in log_10_ scaled) (**Supplementary Figure 2A**) of the nine cell phenotypes assessed within the tissue area ‘AA’, we identified four namely B cells CD21^-^ CD23^-^, B cells CD21^-^ CD23^+^, B cells CD21^+^ CD23^-^ and T cells CD4^+^ which demonstrated significant associations with patient survival, as evidenced by their high concordance indices and significant -log_10_(p-values). Notably, the density of B cells CD21^-^ CD23^-^ and B cells CD21^+^ CD23^-^ exhibited strongest predictive capabilities, with C-index values of 0.57 and 0.58, respectively, and robust statistical significance (p = 0.001 and p = 0.002, respectively). The cellular density of B cells CD21^+^ CD23^+^, of the three different FDC subsets FDC CD21^-^ CD23^+^, FDC CD21^+^ CD23^-^ and FDC CD21^+^ CD23^+^, and of T cells CD8^+^ did not show statistically significant associations with patient survival when the entire tumor area was considered (**Figure 3D** and **Supplementary Table 1**). Interestingly, only two cell phenotypes exhibited a significant prognostic impact in TLS specific areas. The density of B cells CD21^+^ CD23^-^ within the TLS regions remained a significant predictor of patient survival with a C-index of 0.57 despite displaying a slightly lower p-value compared to the ‘AA’ area counterpart. Additionally, FDC CD21^+^ CD23^-^ emerged as a second cell phenotype significantly impacting the survival of patient when located in TLS areas (C-index: 0.58), whereas B cells CD21^-^ CD23^-^, B cells CD21^-^ CD23^+^ and T cells CD4^+^ significant prognostic impact was lost (**Figure 3E** and **Supplementary Table 1**). Finally, we demonstrated that the density of DC-LAMP^+^ cells within TLS was not of prognostic value in this cohort (**Figure 3E** and **Supplementary Table 1**). The survival analysis results of additional parameters such as TLS relative area (unscaled), TLS number (scaled), and of cell densities within different tumor areas are indicated in **Supplementary Table 1**.

In conclusion, our results suggested that both TLS relative area and TLS composition should be considered when evaluating TLS prognostic benefit, both features showing prognostic value. Moreover, while the presence of lymphoid aggregates is frequently associated with an improved prognostic value in multiple cancer indications (8, 9), tools need to be developed to better characterize, score these TLS structures at a tissue level, and evaluate their real impact on the survival of cancer patients.

### TLS score aim and calculation

**Figure 4:**
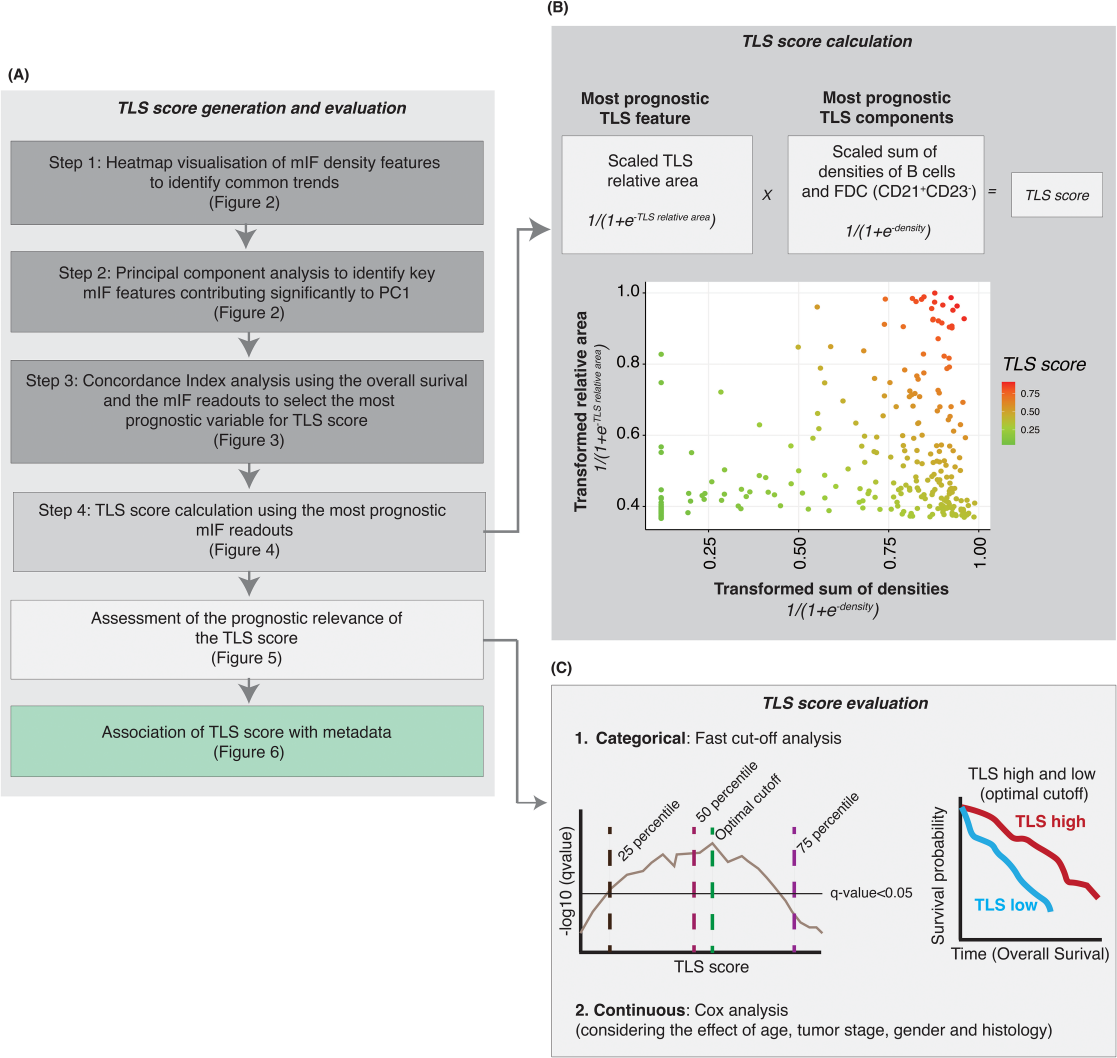
Generation of a TLS score after identifying the most consistently prognostic TLS mIF features. **(A)** Steps performed to identify the key multiplex IF cell phenotypes and TLS features to generate a TLS score with prognostic value. **(B)** Based on the results from heatmap, principal component analysis and survival analysis we selected the most prognostic (i) TLS feature (relative area of the TLS) and (ii) TLS components (densities of B cells and follicular dendritic cells CD21^+^ CD23^-^) to generate a TLS score. The TLS relative area was scaled, and the logistic function was applied. For the CD21^+^ CD23^-^ cells (B cells and FDC), cell densities were scaled and summed followed by transformation of data using the logistic function. The TLS score is the product of (i) and (ii). **(C)** A log-rank test was performed to assess the prognostic relevance of TLS score, at multiple cut-off points, and the optimal cut-off was selected to categorize the NSCLC samples into TLS-high and -low groups. The optimal cut-off was selected based on two criteria – minimizing the FDR-adjusted p-value and balancing the number of samples in the TLS-high and TLS-low categories. Kaplan-Meier survival analysis was performed to assess the survival difference between the TLS-high and TLS-low samples, using the optimal cut-off-based TLS stratification.

In this context, we aimed to generate a TLS tissue scoring system, called TLS Score, which would reflect the TLS biology within the tumor tissue. In many cancer types, TLS maturation status, commonly assessed by DC-LAMP positivity or the presence of CD21^+^ FDC network and a CD23^+^ Germinal Centre, is described as one of the main drivers of TLS prognostic value (8, 9). Although generating a TLS Score based on the TLS knowledge we have from the literature was an attractive option, the TLS biology complexity and different prognostic values of TLS components highlighted in **Figures 2** and **3** led us to generate a data-driven scoring system. **Figure 4A** summarizes the strategy and multi-step process to generate a TLS Score based on our TLS multiplex IF data: step 1- heatmap of mIF density features to identify common trends (**Figure 2A**), step 2- PCA analysis to identify key TLS multiplex IF features (**Figures 2B, C**), step 3- Concordance index analysis to select the most prognostic TLS features (**Figures 3C–E**) and step 4- TLS Score calculation using the most relevant and prognostic multiplex IF readouts.

**Figure 5:**
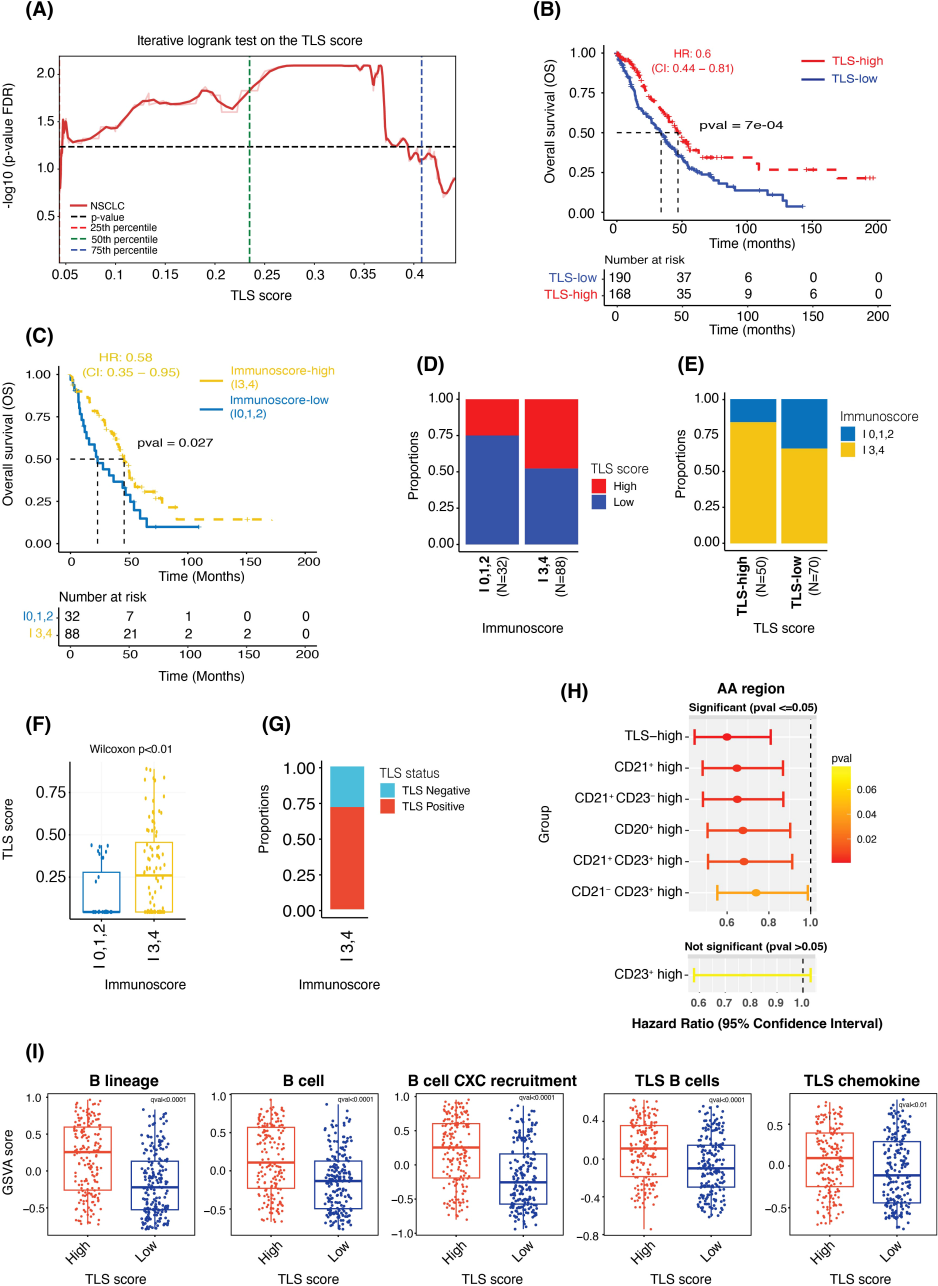
TLS score is prognostic independent of the cutoff used to categorize the data and shows significant association with Immunoscore and gene expression-based signature scores. **(A)** Plot showing FDR-adjusted p-values (from the log-rank test) observed at multiple cut-off points between the TLS-high and TLS-low samples. **(B, C)** Kaplan-Meier survival curves showing the difference in overall survival between the TLS-high and -low groups **(B)** and between the Immunoscore-high (I3,4) and Immunoscore-low (I0,1,2) groups **(C)**. **(D, E)** Bar plots showing the proportions of TLS-high and TLS-low samples in the Immunoscore-high and -low groups **(D)** and the proportions of Immunoscore-high and -low samples in TLS-high and TLS-low groups **(E)**. **(F)** Box plot comparing the TLS Score (as a continuous value) between Immunoscore-high and Immunoscore-low groups. **(G)** Bar plot showing the proportions of TLS-high and TLS-low samples in the Immunoscore-high group. **(H)** A plot showing hazard ratios, the 95% confidence interval of HR and the p-value from the Wald test of TLS-high (vs TLS-low, used as reference), CD21^+^ high (vs CD21^+^ low), CD21^+^ CD23^-^ high (vs CD21^+^ CD23^-^ low), CD20^+^ high (vs CD20^+^ low), CD21^+^ CD23^+^ high (vs CD21^+^ CD23^+^ low), CD21^-^ CD23^+^ high (vs CD21^-^ CD23^+^ low) and CD23^+^ high (vs CD23^+^ low) cases. **(I)** Box plots showing the difference in the gene expression-based enrichment scores of B cells and TLS signatures between the TLS-high and TLS-low groups.

**Figure 6:**
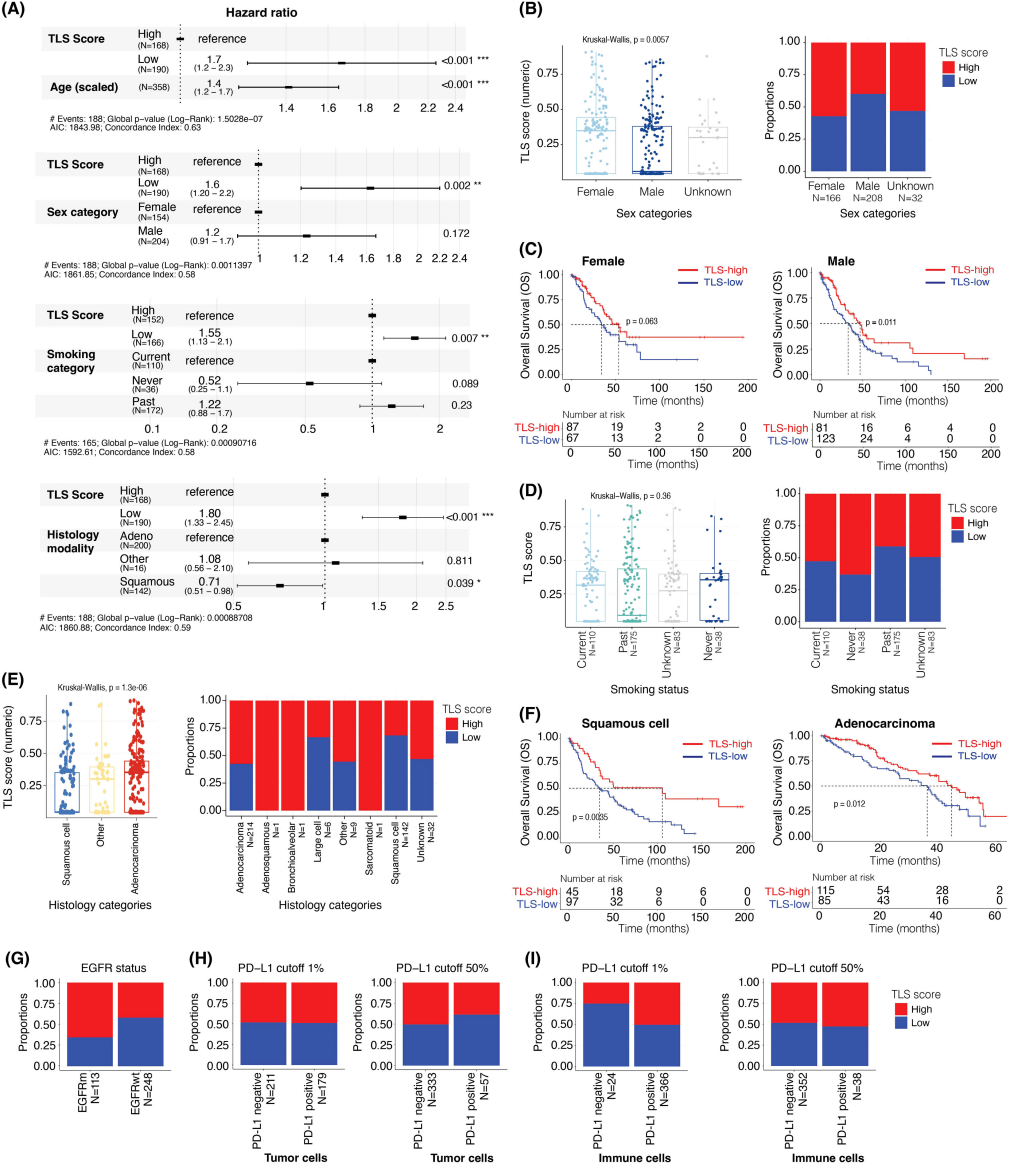
TLS score is prognostic after adjusting for age, sex, smoking status, and is associated with EGFR mutational status but not PD-L1 positivity status. **(A)** The hazard ratios and 95% confidence interval of TLS scores adjusted for the effect of **(i)** age, **(ii)** sex, **(iii)** smoking status, and (iv) histology using multivariable Cox regression analysis and overall survival data. **(B)** Box plot showing the difference in TLS scores (as a continuous value) in male and female samples (left panel). Bar plot showing the proportions of TLS-high and -low samples in male and female (right panel). **(C)** Kaplan-Meier plots showing the difference in overall survival between TLS-high and TLS-low groups in female (left panel) and male (right panel). **(D)** Box plot comparing the TLS scores (as a continuous value) by smoking status (left panel). Bar plot showing the proportions of TLS-high and -low samples in current, never, and past smokers (right panel). **(E)** Box plot comparing the TLS score (as a continuous value) in different histological subtypes of NSCLC (left panel). Bar plot showing the proportions of TLS-high and -low in different histological subtypes of NSCLC samples (right panel). **(F)** Kaplan-Meier plots showing the difference in overall survival between the TLS-high and TLS-low groups in squamous cell carcinoma (left panel) and adenocarcinoma (right panel). **(G)** Bar plot showing the proportions of TLS-high and -low samples in EGFR mutant and wildtype samples. **(H, I)** Bar plots showing the proportions of TLS-high and TLS-low groups in PD-L1 staining positive and negative samples in tumor cells ‘TC’ **(H)** and immune cells ‘IC’ **(I)** at 1% (left panels) or 50% (right panels) PD-L1 positivity cut-offs.

As previously described, the PCA analysis (step 2) revealed that these readouts contained similar information, requiring the selection of optimal readouts for generating a TLS Score to avoid using redundant information (**Figure 2**). The subsequent concordance index analysis conducted aimed to identify the most prognostic TLS features and cell density readouts (**Figure 3** and **Supplementary Table 1**). These analyses highlighted the three most prognostic TLS parameters selected to generate a TLS tissue score (step 3): TLS scaled relative area, B cell CD21^+^ CD23^-^ and FDC CD21^+^ CD23^-^ cell densities. The TLS Score uses (i) the scaled TLS relative area and (ii) the scaled sum of B cells CD21^+^ CD23^-^ and FDC CD21^+^ CD23^-^ densities (in log_10_ scale, see the methods section). It is important to emphasize that in order to consistently generate a TLS Score for all the patients within this cohort and include TLS negative cases for which TLS data were absent (resulting in missing values marked as NA for TLS relative area and cell densities within TLS), we addressed these missing values by treating them as 0. Furthermore, the data underwent a log_10_ transformation for normalization and variance stabilization and scaling to ensure consistent scales for the maximum and minimum values across multiple TLS features. The scaled sum of B cells and FDC densities and the scaled relative area were transformed using a logistic function and then multiplied to generate the TLS score (**Figure 4B**). The calculated TLS Score values ranged from 0 to 1 (**Figure 4B**) and can be used as a categorical or continuous variable. As indicated in **Figure 4C**, we then assessed the TLS Score prognostic relevance through an iterative log-rank test analysis and a univariate Cox analysis which considered the effect other metadata variables such as age, sex, smoking status and histology categories. The results are presented in **Figures 5** and **6** and described below.

### TLS score correlation with immunoscore and TLS gene expression signatures

After the TLS Score generation, we first wanted to evaluate its prognostic impact in NSCLC. The iterative log-rank test, where each cut-off point contained at least 10% of the total observation in each category, demonstrated that the TLS score was significantly prognostic independently of the cut-off used to categorize the data (**Figure 5A**). In the downstream analyses, we hence used the optimal cut-off value of 0.28 to divide the NSCLC cohort (N = 358) into two groups: TLS-high (TLS Score ≥ 0.28) and TLS-low (TLS Score < 0.28) and considered this score as a categorical variable, TLS-high versus -low. Survival analysis between the TLS-high (N = 168) and -low (N = 190) groups indicated that the TLS-high group had a longer overall survival compared to the TLS-low group (median OS of 46.2 months and 33.8 months, respectively, p < 0.001 from log-rank test, HR: 0.6, 95% CI: 0.44 - 0.81) (**Figure 5B**), confirming the prognostic power of this TLS scoring system in NSCLC.

Another immune cell scoring system, known as Immunoscore and which quantifies tumor infiltrating lymphocytes (TILs) using CD3 and CD8 markers, has already proven to be highly valuable in clinic. It has indeed been shown to be more powerful than the traditional staging system and is now considered as a prognostic indicator (1, 2). In this regard, we wanted to confirm the clinical value of the Immunoscore (N = 120) in this cohort and assess if there was any correlation between Immunoscore and TLS score status. Due to the low number of samples in I0 (N = 10), I1 (N = 1), I2 (N = 21), I3 (N = 25) and I4 (N = 63) after considering the survival data, we consolidated the Immunoscore classification into two major groups – Immunoscore-high and Immunoscore-low – to mitigate the sample size challenge. Survival analysis comparing Immunoscore-high group (I3 and I4; N = 88) to Immunoscore-low group (I0, I1, and I2; N = 32) revealed that indeed Immunoscore-high patients had longer overall survival compared to Immunoscore-low patients (median OS of 46.2 months in the -high group versus 23.8 months in the -low group, p < 0.03 from log-rank test; HR: 0.58, 95% CI: 0.35 - 0.95) (**Figure 5C**). Overall, these results showed that both TLS Score and Immunoscore -high values were associated with better survival. Additional univariate Cox analysis confirmed these observations, greater and significant hazard ratios being obtained for both TLS-high and Immunoscore-high groups compared to their respective -low categories (**Supplementary Figures 3A, B**).

Next, we evaluated the association between TLS score and Immunoscore. Despite observing a significant increase in the proportion of TLS-high samples in the Immunoscore-high compared to the -low samples, only 53% of the Immunoscore-high samples were also TLS-high (**Figure 5D**). Conversely, high proportions of Immunoscore-high were found in both TLS-high and TLS-low cases. Immunoscore-high represented indeed 83% of the TLS-high and 65% of the TLS-low cohorts (**Figure 3E**). These results suggested that despite observing a positive trend between the Immuno- and TLS- scores, there was no absolute concordance between these two scoring systems, possibly due to the fact that they are primarily based on two different immune cell populations, T cells and B cells, respectively. These findings were further confirmed in **Figures 5F, G**, where a high range of TLS Score values was observed within Immunoscore-high samples (**Figure 5F**), with more than 25% of these samples being TLS negative (**Figure 5G**).

Multiple histology methods and assays are used to detect TLS and assess their maturity levels, including single IHC assays for the CD20, CD21 and CD23 markers. In this regard, we wanted to assess the prognostic value of the TLS Score, which considers multiple TLS features based on the positivity status of these markers taken all together and compare it with the prognostic value of each marker considered as a single marker, as we would do for single IHC assays data. As presented in **Figure 5H**, we calculated the total density of positive cells for single markers (CD20, CD21 and CD23) and the combined markers for CD21 and CD23 (CD21^+^ CD23^-^, CD21^-^ CD23^+^, and CD21^+^ CD23^+^) located within the entire tumor area ‘AA’. For each density results, the cohort was divided into two groups, high and low density based on the median cut-off. For TLS Score classification, we used the previously described optimal cut-off. Subsequently, we conducted univariate Cox analyses to assess the hazard ratio, measuring the relative risk of death, between (i) density-high versus density-low (reference) category for each individual marker, (ii) TLS-high versus -low (reference) category and (iii) density-high versus density-low (reference) group for combined markers CD21^+^ CD23^-^, CD21^-^ CD23^+^, and CD21^+^ CD23^+^. Outstandingly, the TLS-high group demonstrated the most pronounced and significant impact on survival outcomes, confirming the added value of the TLS tissue score. No significant result was obtained for the CD23^+^ density high, probably due to the lower prevalence of these cells within the tumor tissues.

Another way commonly used to assess TLS is by evaluating gene expression (GE) signatures specific to TLS. Thus, we first wanted to assess how our TLS Score correlated with the two main and commonly used ‘12-chemokine TLS’ and ‘TLS T_H_1 B cell’ GE signatures (8) (N = 375 samples). The 12-chemokine TLS signature includes *CCL2, CCL3, CCL4, CCL5, CCL8, CCL18, CCL19, CCL21, CXCL9, CXCL10, CXCL11* and *CXCL13* genes (12/12 genes overlap with our GE data), and TLS T_H_1 B cell signature contains *CD4, CCR5, CXCR3, CSF2, IGSF6, IL2RA, CD38, CD40, CD5, MS4A1, SDC1, GFI1, IL1R1, IL1R2, IL10, CCL20, IRF4, TRAF6* and *STAT5A* genes (15/19 genes overlap, *IGSF6, SDC1, GFI1* and *STAT5A* genes were missing). B cells being the major TLS immune component, we also assessed the expression of multiple B-cell gene signatures, named ‘B cell’ (*CD19, MS4A1, CD22* and *CD79A* genes) (32), ‘B lineage’ (*CD19, MS4A1, CD22, CD79A* and *CXCL13* genes) and ‘B cell CXC recruitment’ (*CD19, MS4A1, CD22, CD79A* and *CXCL13*). Using gene set variation analysis (GSVA), we observed a significant difference in the enrichment of these five signatures when comparing TLS-high with TLS-low tissues (**Figure 5I**), but also when comparing TLS negative samples to TLS positives cases (**Supplementary Figure 3C**). Indeed, while TLS-high and TLS positive tissues demonstrated higher GSVA scores for all GE signatures, TLS-low and TLS negative samples showed a lower GSVA scores.

Together, these results confirmed the prognostic relevance of the Immunoscore in NSCLC but also demonstrated the clinical potential of this TLS Score, which was associated with an improved overall survival and was positively associated with published TLS gene expression signatures.

### TLS score association with clinical and demographical parameters

Next, we aimed to determine the relationships between TLS Score and clinicopathological features of the overall population. We performed Cox regression analyses to investigate the prognostic relevance of TLS score, along with several other variables including age, sex category, smoking status, and NSCLC histology. However, due to a substantial amount of missing data overlapping for sex, smoking status, and histology categories, we were unable to include all these variables in a single survival model.

Consequently, we employed four distinct survival models to assess the prognostic value of the TLS Score while adjusting for the impact of the following variables: (i) age, (ii) sex, (iii) smoking status, and (iv) histology. Additionally, we examined the association between the TLS groups (-high and -low) and other covariates such as *EGFR* (Epidermal Growth Factor Receptor) mutational status and PD-L1 (Programmed Death-Ligand 1) positivity status.

In **Figure 6A**, we showed that although the age of the patient had a significant impact on the overall survival (N = 358) (HR: 1.42, 95% CI: 1.21 – 1.67, p < 0.001, with age scaled data), this did not affect the prognostic value of the TLS Score, with a hazard ratio of 0.32 (95% CI: 0.16 – 0.63, p < 0.001). Moreover, even when considering the sex category (N = 358), smoking status (N = 318) and NSCLC histology (N = 358) factors, the TLS Score impact on the overall survival remained significant (**Figure 6A**). Interestingly, the female category exhibited a significantly higher TLS Score than the male category, with TLS score median values of 0.35 and 0.05, respectively (Kruskal-Wallis, p = 0.0057) (**Figure 6B**, left panel). This could be explained by higher proportion of TLS-high cases in the Female category, as they represented approximately 68% of the female population, whereas this number fell at 40% in the male population (**Figure 6B**, right panel). We then segregated the two gender categories into two sub-groups according to their TLS Score status, TLS-high or TLS-low, to assess how this score impacted the overall survival in each group. Whereas the median OS increased by 19.3 months in the female category (**Figure 6C**, left panel) and by 14.1 months in the male category (**Figure 6C**, right panel) when we compared TLS-high with TLS-low cases, only the male group result was significant (p = 0.011), the female group describing a p-value of 0.063.

When considering the smoking status, the TLS Score still had a significant association with survival (HR: 0.38, 95% CI: 0.19 – 0.77, p = 0.008) while the impact of the smoking category was not significant in this analysis (**Figure 6A**). Furthermore, although we did not observe a statistically significant relationship between the TLS Score value and the smoking categories (Kruskal-Wallis, p = 0.0057) (**Figure 6D**, left panel), the proportion of TLS-high cases tended to be higher in patients who never smoked at the time of the survey (63%), compared to the current (52%) and past smokers (43%) (**Figure 6D**, right panel).

We then evaluated the impact of NSCLC histology on the overall patient survival and TLS Score repartition within different lung cancer sub-types. This cohort contains 214 adenocarcinoma, 1 adenosquamous, 1 bronchioalveolar, 1 large cell, 142 squamous cell, 1 sarcomatoid, 9 “other”, and 29 “unknown” cases. Due to the low number of tissues in some categories, we focused on the adenocarcinoma (N = 214) and squamous cell (N = 142) categories for the subsequent analyses. In **Figure 6A**, we observed a significant impact of the histological modality, the squamous cell category reducing the risk of death by 28% (HR: 0.72, 95% CI: 0.52 – 0.99, p = 0.043) compared to the adenocarcinoma category when taken as reference. Furthermore, considering the histology type and TLS Score as covariate did not impact the prognostic relevance of this tissue score which described a hazard ratio of 0.27 (95% CI: 0.14 – 0.56, p < 0.001) (**Figure 6A**). Interestingly, we observed lower TLS Score values in the squamous cell category than in the adenocarcinoma category, with TLS score median values of 0.04 and 0.37, respectively (Kruskal-Wallis, p = 1.3e-06) (**Figure 6E**, left panel). This could be explained by a lower proportion of TLS-high cases in the squamous cell compared to the adenocarcinoma subtype, with 30% of squamous cell and 58% of adenocarcinoma cases being TLS-high (**Figure 6E**, right panel). A high TLS Score was however associated with a better outcome in both categories (**Figure 6F**). Indeed, when comparing TLS-high with TLS-low cases, the median OS was increased by 70.5 months in squamous cell (34.2 vs 104.7 months, p = 0.0035) (**Figure 6F**, left panel), and by 8.3 months in adenocarcinoma (35.4 vs 43.7 months, p = 0.012) (**Figure 6F**, right panel).

These NSCLC cases have also been characterized for *EGFR* mutation status and PD-L1 expression levels. We thus wanted to assess the prevalence of TLS-high and TLS-low cases within each drug segment category. Interestingly, approximately 66% of the *EGFR* mutant samples (N = 113) were TLS-high against 43% of the *EGFR* wild-type cohort (N = 248) (**Figure 6G**), suggesting an impact of the mutational status on the TLS Score value (hypergeometric test, qval <0.001, **Supplementary Figure 3D**).

Finally, we evaluated the TLS Score repartition according to PD-L1 categories characterized by different PD-L1 positivity cut-off (>1% or >50% positivity) on the surface of the tumor cells (**Figure 6H**) or immune cells (**Figure 6I**). These categories were as follow: PD-L1 1% TC (PD-L1 cut-off >1% on tumor cells) (**Figure 6H**, left panel), PD-L1 50% TC (PD-L1 cut-off >50% on tumor cells) (**Figure 6H**, right panel), PD-L1 1% IC (PD-L1 cut-off >1% on immune cells) (**Figure 6I**, left panel) and PD-L1 50% IC (PD-L1 cut-off >50% on immune cells) (**Figure 6I**, right panel).

We did not observe a significant impact of PD-L1 status on the TLS Score proportions, approximately 50% of cases describing TLS-high scores in all the PD-L1 positive cohorts and independently of the PD-L1 cut-off used (**Figures 6H, I**). However, it is interesting to note that change in this cut-off had a slight impact on the TLS Score proportion within PD-L1-negative cohorts, TLS-high cases representing 50% of the PD-L1-negative cases among the PD-L1 50% IC cohort and 25% among the PD-L1 1% IC cohort (**Figure 6I**). We found that the TLS score is prognostic after adjusting for PD-L1 status in tumor and immune cells. (**Supplementary Figure 4**). As a summary, although this analysis did not demonstrate any impact of the PD-L1 status on the TLS Score, it highlighted the importance of keeping the same analysis cut-off across studies and when comparing data.

## Discussion

TLS are commonly associated with favorable prognosis in many cancer types. However, conflicting studies suggest that not all aggregates are functionally equivalent and minimal characteristics may be required to form a functional TLS (8–13). Moreover, variable numbers of TLS can be present within a tumor tissue, each one of these structures describing unique characteristics (such as the size, cellular composition, location, maturation stage), each feature having a potential impact on the collective clinical power. In this context, we aimed at evaluating the prognostic value of TLS in NSCLC by establishing a TLS Score that captures the diversity of TLS within a tumor, considering functional and compositional features.

We observed a large variety of TLS and B-cell aggregates within tumor tissues, each differing in size, organization level and cellular composition. Interestingly, high density of CD21^+^ and CD23^+^ cells, markers considered as TLS maturity markers, were observed in both small and large aggregates, suggesting that a high degree of TLS organization and maturation is independent of aggregate size.

Based on our observations and the image analysis readouts, we considered multiple ways for calculating a tissue score capturing TLS compositional, functional, and organizational diversity within tissues. We initially considered (i) TLS relative area and (ii) the density of each cell phenotype, for each TLS, and combined these data into a unique TLS tissue score. However, this idea was challenged by the Concordance index survival analysis which demonstrated a prognostic significance of only specific cells within TLS, particularly B cells and FDC CD21^+^ CD23^-^. A parallel can be made with the three TLS maturation stages first identified by Karīna Siliņa et al. in human lung squamous cell carcinoma, which are: (i) early TLS (CD21^-^ CD23^-^); (ii) primary follicle-like TLS with differentiated FDC (CD21^+^ CD23^-^); (iii) and mature secondary follicle-like TLS with a germinal center reaction (CD21^+^ CD23^+^) (10). Moreover, the germinal center reaction, crucial for B cell activation and differentiation, has demonstrated significant relevance for patient survival in various cancer types (8–10, 33–37). In lymph nodes in mice, the long-term retention of antigens in germinal centers is controlled by the spatial organization of the follicular dendritic cell network and notably high levels of CD21 expression on their surface (38), thus supporting our findings.

The other cell phenotypes assessed did not show significant impact when located within TLS, thus questioning the relevance of including them into a TLS scoring system which aimed at evaluating the clinical value of these structures. In light of these findings, we refined our TLS Score calculation strategy to generate a data-driven score, that would only include the readouts identified as the most prognostic and robust. The final score was calculated using (i) the scaled sum of B cells and FDC CD21^+^ CD23^-^ densities (log_10_), and (ii) the TLS scaled relative area, which were the three most prognostically relevant and robust TLS features in this NSCLC cohort.

Excitingly, this TLS Score demonstrated a strong prognostic power, independently of the cut-off used, and added value over the commonly used TLS markers CD20, CD21 and CD23 when assessed as single markers in the context of single IHC analysis. These results highlighted the relevance of using combination of markers specific to TLS, such as a TLS tissue score, instead of single markers, to accurately evaluate how the TLS biology and heterogeneity within a tumor tissue impact patients’ prognosis. Nevertheless, this score being generated based on the specific detection of TLS structures in tumor resections, it would not solve the challenge of TLS detection and assessment in tumor biopsies, fewer number of TLS or none being detected due to the small size of the tumor cores.

We could also question the reproducibility of the TLS Score values and subsequent prognostic results if different tissue sections of the same tumors were stained. Small TLS observed in one section could indeed correspond to larger TLS cut near the surface and thus displaying different sizes, maturation status and cellular composition – parameters used to calculate the TLS Score values. In this context, it would be relevant to further explore the TME organization in tissues TLS-high compared to TLS-low cases with the aim of highlighting TLS TME spatial signatures that could be used as TLS surrogate in tumor biopsies. We could for example describe the spatial characteristics of areas outside TLS, understand the distribution of immune cells within the tissue, how they interact with each other and with tumor cells. In this regard, we assessed if there was any correlation between TLS Score and Immunoscore, another tissue scoring system focusing on the T cell population using CD3 and CD8 markers and now considered as a prognostic indicator in multiple tumor types (1, 2). While we demonstrated higher TLS Score values in Immunoscore-high cases overall, in agreement with the literature reporting that a high CD8^+^ T cell infiltration is significantly correlated with the presence of mature TLS (5, 8, 9, 39–43), this result should be interpreted carefully and would require additional investigations, since one quarter of the Immunoscore-high cases were TLS-negative. We could thus hypothesize that this T cell sub-population might not be the optimal one to be considered as a TLS surrogate, and evaluating other cell populations or combinations of different cell types as potential TLS surrogates is necessary. We could assess the distribution of another T cell sub-population, such as CD4^+^ T cells, but also myeloid cells, such as dendritic cells, macrophages, or lymphatic vessels and high endothelial venules for which increased densities have been described in TLS-positive tissues (8, 13, 44–47). Additionally, it is important to note the lack of Immunoscore data for 286 out of 406 NSCLC cases, which might affect these results.

Genomic technologies are another common way to evaluate TLS presence within tumor tissues in the clinic, using gene expression (GE) signatures specific to TLS, the main two being the ‘12-chemokine signature’ and ‘T_H_1 cell and B cell signature’ first published by Sautès-Fridman et al. (8, 33, 48). GSVA analyses revealed significantly higher TLS GE signature scores in TLS-high compared to TLS-low cases, despite a large proportion of samples having low signature score, thus supporting the clinical potential of this TLS Score. One possible explanation for the low GSVA scores obtained could be the fact that transcriptomic data were generated using tumor bulk tissues, whereas the TLS Score was based on multiplex IF data specific to the TLS structures within the tumor and hence reflected the power of spatial over tumor bulk technologies. Furthermore, TLS GE signatures are related to either chemokines or cell populations involved in TLS neogenesis and considered as pan-cancer signatures (8); hence, refining TLS GE signatures with the support of spatial transcriptomic technologies, or defining new ones that show greater specificity to the TLS maturation degrees might be of relevance to improve our understanding of TLS impact in the clinic. Another explanation could be related to the fact that this TLS Score has been developed based on multiplex IF data obtained from one cohort of NSCLC patients which includes different histology types, tumor stages, and demographical characteristics, with missing information for many patients. Thus, we cannot exclude the fact that the TLS Score results obtained, and its promising prognostic value may be specific of one sub-category of the NSCLC patient population. It will thus be necessary to confirm the impact of this scoring system in other lung cancer cohorts as well as in different tumor indications.

These observations about the NSCLC cohort, which can be considered as limitations, are particularly relevant and should be taken into account when interpreting the results assessing whether clinical and demographical features such as age, sex, smoking status, histology category, *EGFR* mutational and PD-L1 positivity status contributed to TLS heterogeneity and correlated with our TLS Score. We indeed demonstrated that the TLS Score prognostic value was independent of age, sex, smoking category, and histology modality features. Besides, we revealed a higher prevalence of TLS-high cases in (i) the female compared to the male category, (ii) the adenocarcinoma compared to the squamous cell subtype, and (iii) *EGFR* mutant compared to *EGFR* wild-type samples. No correlation with PD-L1 expression levels was observed. Interestingly, and in coherence with our findings, a study reported a higher frequency of *EGFR* mutations in tumors enriched with mature dendritic cells, cell subset considered as a hallmark of TLS (49). A parallel can also be made with a study which highlighted a predictive value of mature TLS to immune checkpoint inhibitor in solid tumors, independently of PD-L1 expression (5). In contrast, multiple studies evaluating the impact of TLS in lung adenocarcinoma did not find a correlation between TLS density and maturity and features such as age, sex, *EGFR* mutation, pathological types or smoking status (50–52).

Furthermore, these NSCLC cases being baseline tumors coming from patients having received chemotherapy or radiotherapy treatments, a fundamental next step would be to evaluate the clinical value of this TLS Score for patients treated with immunotherapies. The identification of reliable predictive biomarkers of response to immunotherapies is indeed a current unmet medical need. In this context, Vanhersecke et al. recently demonstrated that the presence of mature TLS CD23^+^ is predictive of response to immunotherapies in multiple tumor types, independently of PD-L1 expression status and CD8^+^ T cell density (5). The calculation of the TLS Score relying on the density of cells composing the germinal center, which is a feature characterizing mature TLS, evaluating and comparing the predictive values of (i) TLS Score and (ii) presence of mature TLS would inform us further on the clinical benefit of combining multiple TLS features into one unique tissue score.

In conclusion, we developed and demonstrated the prognostic value of a TLS tissue score in NSCLC which allows a better representation and characterization of the TLS biology and heterogeneity undergoing within a tumor. Our aim being the identification of biomarkers which could be used in the clinic to select patients who are more likely to benefit from immunotherapies, the next step is to evaluate the predictive power of this TLS Score in cohorts of patients treated with immune checkpoint inhibitors. Finally, this TLS scoring system could be used as a tool to assess how TLS impact the organization of the tumor microenvironment, thus supporting the discovery of TLS TME spatial biomarkers, surrogates of mature TLS, to help overcome the challenge of TLS detection and assessment in tumor biopsies in the clinic.

## Data Availability

The original contributions presented in the study are included in the article/**Supplementary Material**, further inquiries can be directed to the corresponding author.

## References

[1] Galon J Mlecnik B Bindea G Angell HK Berger A Lagorce C. Towards the introduction of the ‘Immunoscore’ in the classification of Malignant tumours. J Pathol. (2014) 232:199–209. doi: 10.1002/path.4287 24122236 PMC4255306

[2] Bruni D Angell HK Galon J. The immune contexture and Immunoscore in cancer prognosis and therapeutic efficacy. Nat Rev Cancer. (2020) 20:662–80. doi: 10.1038/s41568-020-0285-7 32753728

[3] Bai R Lv Z Xu D Cui J. Predictive biomarkers for cancer immunotherapy with immune checkpoint inhibitors. biomark Res. (2020) 8:34. doi: 10.1186/s40364-020-00209-0 32864131 PMC7450548

[4] Cabrita R Lauss M Sanna A Donia M Skaarup Larsen M Mitra S. Tertiary lymphoid structures improve immunotherapy and survival in melanoma. Nature. (2020) 577:561–5. doi: 10.1038/s41586-019-1914-8 31942071

[5] Vanhersecke L Brunet M Guegan JP Rey C Bougouin A Cousin S. Mature tertiary lymphoid structures predict immune checkpoint inhibitor efficacy in solid tumors independently of PD-L1 expression. Nat Cancer. (2021) 2:794–802. doi: 10.1038/s43018-021-00232-6 35118423 PMC8809887

[6] Helmink BA Reddy SM Gao J Zhang S Basar R Thakur R. B cells and tertiary lymphoid structures promote immunotherapy response. Nature. (2020) 577:549–55. doi: 10.1038/s41586-019-1922-8 PMC8762581 31942075

[7] Petitprez F de Reynies A Keung EZ Chen TW Sun CM Calderaro J. B cells are associated with survival and immunotherapy response in sarcoma. Nature. (2020) 577:556–60. doi: 10.1038/s41586-019-1906-8 31942077

[8] Sautès-Fridman C Petitprez F Calderaro J Fridman WH. Tertiary lymphoid structures in the era of cancer immunotherapy. Nat Rev Cancer. (2019) 19:307–25. doi: 10.1038/s41568-019-0144-6 31092904

[9] Fridman W Meylan M Petitprez F Sun CM Italiano A Sautès-Fridman C. B cells and tertiary lymphoid structures as determinants of tumour immune contexture and clinical outcome. Nat Rev Clin Oncol. (2022) 19:441–57. doi: 10.1038/s41571-022-00619-z 35365796

[10] Silina K Soltermann A Attar FM Casanova R Uckeley ZM Thut H. Germinal centers determine the prognostic relevance of tertiary lymphoid structures and are impaired by corticosteroids in lung squamous cell carcinoma. Cancer Res. (2018) 78:1308–20. doi: 10.1158/0008-5472.CAN-17-1987 29279354

[11] Kroeger DR Milne K Nelson BH. Tumor-infiltrating plasma cells are associated with tertiary lymphoid structures, cytolytic T-cell responses, and superior prognosis in ovarian cancer. Clin Cancer Res. (2016) 22:3005–15. doi: 10.1158/1078-0432.CCR-15-2762 26763251

[12] Koti M Xu AS Ren KYM Visram K Ren R Berman DM. Tertiary lymphoid structures associate with tumour stage in urothelial bladder cancer. Bladder Cancer. (2017) 3:259–67. doi: 10.3233/BLC-170120 PMC5676768 29152550

[13] Yamaguchi K Ito M Ohmura H Hanamura F Nakano M Tsuchihashi K. Helper T cell-dominant tertiary lymphoid structures are associated with disease relapse of advanced colorectal cancer. Oncoimmunology. (2020) 9:1724763. doi: 10.1080/2162402X.2020.1724763 32117589 PMC7028340

[14] Scorer P Scott M Lawson N Ratcliffe MJ Barker C Rebelatto MC. Consistency of tumor and immune cell programmed cell death ligand-1 expression within and between tumor blocks using the VENTANA SP263 assay. Diagn Pathology. (2018) 13:47. doi: 10.1186/s13000-018-0725-9 PMC6058354 30041679

[15] Pagès F Mlecnik B Marliot F Bindea G Ou F-S Bifulco C. International validation of the consensus Immunoscore for the classification of colon cancer: a prognostic and accuracy study. Lancet. (2018) 391:2128–39. doi: 10.1016/S0140-6736(18)30789-X 29754777

[16] Segerer FJ Nekolla K Rognoni L Kapil A Schick M Angell HK. Novel deep learning approach to derive cytokeratin expression and epithelium segmentation from DAPI (2022). Available online at: https://arxivorg/abs/220808284.

[17] Lê S Josse J Husson F. FactoMineR: an R package for multivariate analysis. J Stat Software. (2008) 25:1–18. doi: 10.18637/jss.v025.i01

[18] Hänzelmann S Castelo R Guinney J. GSVA: gene set variation analysis for microarray and RNA-Seq data. BMC Bioinf. (2013) 14. doi: 10.1186/1471-2105-14-7 PMC3618321 23323831

[19] Tu E McGlinchey K Wang J Martin P Ching SL Floc’h N. Anti-PD-L1 and anti-CD73 combination therapy promotes T cell response to EGFR-mutated NSCLC. JCI Insight. (2022) 7. doi: 10.1172/jci.insight.142843 PMC8855814 35132961

[20] Davidson-Pilon C. lifelines: survival analysis in Python. J Open Source Software. (2019) 4. doi: 10.21105/joss.01317

[21] Therneau T. A Package for Survival Analysis in R. R package version 3.7-0, (2024). Available at: https://CRAN.Rproject.org/package=survival.

[22] Alboukadel K. survminer: Drawing Survival Curves using ‘ggplot2’. R package version 0.4.9. (2016) Available at: https://cran.r-project.org/web/packages/survminer.

[23] Harrell FE Lee KL Mark DB. Multivariable prognostic models: issues in developing models, evaluating assumptions and adequacy, and measuring and reducing errors. Stat Med. (1995) 15:361–87. 10.1002/(SICI)1097-0258(19960229)15:4<361::AID-SIM168>3.0.CO;2-4 8668867

[24] Kurshumliu F Sadiku-Zehri F Qerimi A Vela Z Jashari F Bytyci S. Divergent immunohistochemical expression of CD21 and CD23 by follicular dendritic cells with increasing grade of follicular lymphoma. World J Surg Oncol. (2019) 17:115. doi: 10.1186/s12957-019-1659-8 31269981 PMC6610797

[25] Suryani S Fulcher DA Santner-Nanan B Nanan R Wong M Shaw PJ. Differential expression of CD21 identifies developmentally and functionally distinct subsets of human transitional B cells. Blood. (2010) 115:519–29. doi: 10.1182/blood-2009-07-234799 19965666

[26] Allen CD Cyster JG. Follicular dendritic cell networks of primary follicles and germinal centers: phenotype and function. Semin Immunol. (2008) 20:14–25. doi: 10.1016/j.smim.2007.12.001 18261920 PMC2366796

[27] Dieu-Nosjean MC Goc J Giraldo NA Sautes-Fridman C Fridman WH. Tertiary lymphoid structures in cancer and beyond. Trends Immunol. (2014) 35:571–80. doi: 10.1016/j.it.2014.09.006 25443495

[28] Le Rochais M Hemon P Ben-Guigui D Garaud S Le Dantec C Pers JO. Deciphering the maturation of tertiary lymphoid structures in cancer and inflammatory diseases of the digestive tract using imaging mass cytometry. Front Immunol. (2023) 14:1147480. doi: 10.3389/fimmu.2023.1147480 37143660 PMC10151544

[29] Italiano A Bessede A Pulido M Bompas E Piperno-Neumann S Chevreau C. Pembrolizumab in soft-tissue sarcomas with tertiary lymphoid structures: a phase 2 PEMBROSARC trial cohort. Nat Med. (2022) 28:1199–206. doi: 10.1038/s41591-022-01821-3 35618839

[30] Levesque MC St. Clair EW. B cell–directed therapies for autoimmune disease and correlates of disease response and relapse. J Allergy Clin Immunol. (2008) 121:13–21. doi: 10.1016/j.jaci.2007.11.030 18206502

[31] Chung JB Sater RA Fields ML Erikson J Monroe JG. CD23 defines two distinct subsets of immature B cells which differ in their responses to T cell help signals. Int Immunol. (2002) 14:157–66. doi: 10.1093/intimm/14.2.157 11809735

[32] Danaher P Warren S Dennis L D’Amico L White A Disis ML. Gene expression markers of Tumor Infiltrating Leukocytes. J Immunother Cancer. (2017) 5:18. doi: 10.1186/s40425-017-0215-8 28239471 PMC5319024

[33] Sautès-Fridman C Verneau J Sun C Moreira M Chen T Meylan M. Tertiary Lymphoid Structures and B cells: Clinical impact and therapeutic modulation in cancer. Semin Immunol. (2020) 48. doi: 10.1016/j.smim.2020.101406 33248905

[34] Hayashi Y Makino T Sato E Ohshima K Nogi Y Kanemura T. Density and maturity of peritumoral tertiary lymphoid structures in oesophageal squamous cell carcinoma predicts patient survival and response to immune checkpoint inhibitors. Br J Cancer. (2023) 128:2175–85. doi: 10.1038/s41416-023-02235-9 PMC10241865 37016103

[35] Liang H Zhang Z Guan Z Zheng S Lou J Liu W. Follicle-like tertiary lymphoid structures: A potential biomarker for prognosis and immunotherapy response in patients with laryngeal squamous cell carcinoma. Front Immunol. (2023) 14:1096220. doi: 10.3389/fimmu.2023.1096220 36776859 PMC9912937

[36] Lynch KT Young SJ Meneveau MO Wages NA Engelhard VH Slingluff CL Jr.. Heterogeneity in tertiary lymphoid structure B-cells correlates with patient survival in metastatic melanoma. J Immunother Cancer. (2021) 9. doi: 10.1136/jitc-2020-002273 PMC8190052 34103353

[37] He M He Q Cai X Liu J Deng H Li F. Intratumoral tertiary lymphoid structure (TLS) maturation is influenced by draining lymph nodes of lung cancer. J Immunother Cancer. (2023) 11. doi: 10.1136/jitc-2022-005539 PMC10124324 37072348

[38] Martinez-Riano A Wang S Boeing S Minoughan S Casal A Spillane KM. Long-term retention of antigens in germinal centers is controlled by the spatial organization of the follicular dendritic cell network. Nat Immunol. (2023) 24:1281–94. doi: 10.1038/s41590-023-01559-1 PMC7614842 37443283

[39] Gao J Navai N Alhalabi O Siefker-Radtke A Campbell MT Tidwell RS. Neoadjuvant PD-L1 plus CTLA-4 blockade in patients with cisplatin-ineligible operable high-risk urothelial carcinoma. Nat Med. (2020) 26:1845–51. doi: 10.1038/s41591-020-1086-y PMC9768836 33046869

[40] Hiraoka N Ino Y Yamazaki-Itoh R. Tertiary lymphoid organs in cancer tissues. Front Immunol. (2016) 7:244. doi: 10.3389/fimmu.2016.00244 27446075 PMC4916185

[41] Goc J Germain C Vo-Bourgais TK Lupo A Klein C Knockaert S. Dendritic cells in tumor-associated tertiary lymphoid structures signal a Th1 cytotoxic immune contexture and license the positive prognostic value of infiltrating CD8+ T cells. Cancer Res. (2014) 74:705–15. doi: 10.1158/0008-5472.CAN-13-1342 24366885

[42] Behr DS Peitsch WK Hametner C Lasitschka F Houben R Schönhaar K. Prognostic value of immune cell infiltration, tertiary lymphoid structures and PD-L1 expression in Merkel cell carcinomas. Int J Clin Exp Pathology. (2014) 7:7610–21. PMC4270630 25550797

[43] Di Caro G Bergomas F Grizzi F Doni A Bianchi P Malesci A. Occurrence of tertiary lymphoid tissue is associated with T-cell infiltration and predicts better prognosis in early-stage colorectal cancers. Clin Cancer Res. (2014) 20:2147–58. doi: 10.1158/1078-0432.CCR-13-2590 24523438

[44] Kang W Feng Z Luo J He Z Liu J Wu J. Tertiary lymphoid structures in cancer: the double-edged sword role in antitumor immunity and potential therapeutic induction strategies. Front Immunol. (2021) 12:689270. doi: 10.3389/fimmu.2021.689270 34394083 PMC8358404

[45] Han J Dong L Wu M Ma F. Dynamic polarization of tumor-associated macrophages and their interaction with intratumoral T cells in an inflamed tumor microenvironment: from mechanistic insights to therapeutic opportunities. Front Immunol. (2023) 14:1160340. doi: 10.3389/fimmu.2023.1160340 37251409 PMC10219223

[46] Bobik A Kyaw TS Tipping P Toh BH. M1 macrophages, key contributors to lymphoid neogenesis in atherosclerotic aorta. Cardiovasc Res. (2014) 101:339–41. doi: 10.1093/cvr/cvu019 24469535

[47] Koscsó B Kurapati S Rodrigues RR Nedjic J Gowda K Shin C. Gut-resident CX3CR1hi macrophages induce tertiary lymphoid structures and IgA response in situ. Sci Immunol. (2020) 5. 10.1126/sciimmunol.aax0062 PMC7296464 32276965

[48] Li R Berglund A Zemp L Dhillon J Putney R Kim Y. The 12-CK score: global measurement of tertiary lymphoid structures. Front Immunol. (2021) 12:694079. doi: 10.3389/fimmu.2021.694079 34267760 PMC8276102

[49] Biton J Mansuet-Lupo A Pecuchet N Alifano M Ouakrim H Arrondeau J. TP53, STK11, and EGFR mutations predict tumor immune profile and the response to anti-PD-1 in lung adenocarcinoma. Clin Cancer Res. (2018) 24:5710–23. doi: 10.1158/1078-0432.CCR-18-0163 29764856

[50] Xu X Gao Y Duan S Ding Q Wang X Dai X. Clinical implications and molecular features of tertiary lymphoid structures in stage I lung adenocarcinoma. Cancer Med. (2023) 12:9547–58. doi: 10.1002/cam4.5731 PMC10166963 36880167

[51] Ren F Xie M Gao J Wu C Xu Y Zang X. Tertiary lymphoid structures in lung adenocarcinoma: characteristics and related factors. Cancer Med. (2022) 11:2969–77. doi: 10.1002/cam4.4796 PMC9359870 35801360

[52] Domblides C Rochefort J Riffard C Panouillot M Lescaille G Teillaud JL. Tumor-associated tertiary lymphoid structures: from basic and clinical knowledge to therapeutic manipulation. Front Immunol. (2021) 12:698604. doi: 10.3389/fimmu.2021.698604 34276690 PMC8279885

