# Supplementary material for: Exploring the impact of tertiary lymphoid structures maturity in NSCLC: insights from TLS scoring
Source: Front Immunol. 2024 Sep 20;15:1422206. doi: 10.3389/fimmu.2024.1422206 (PMC11457083; doi:10.3389/fimmu.2024.1422206)
Supplement: Supplementary file 1 [file DataSheet1.pdf]

SUPPLEMENTAL FIGURE 1

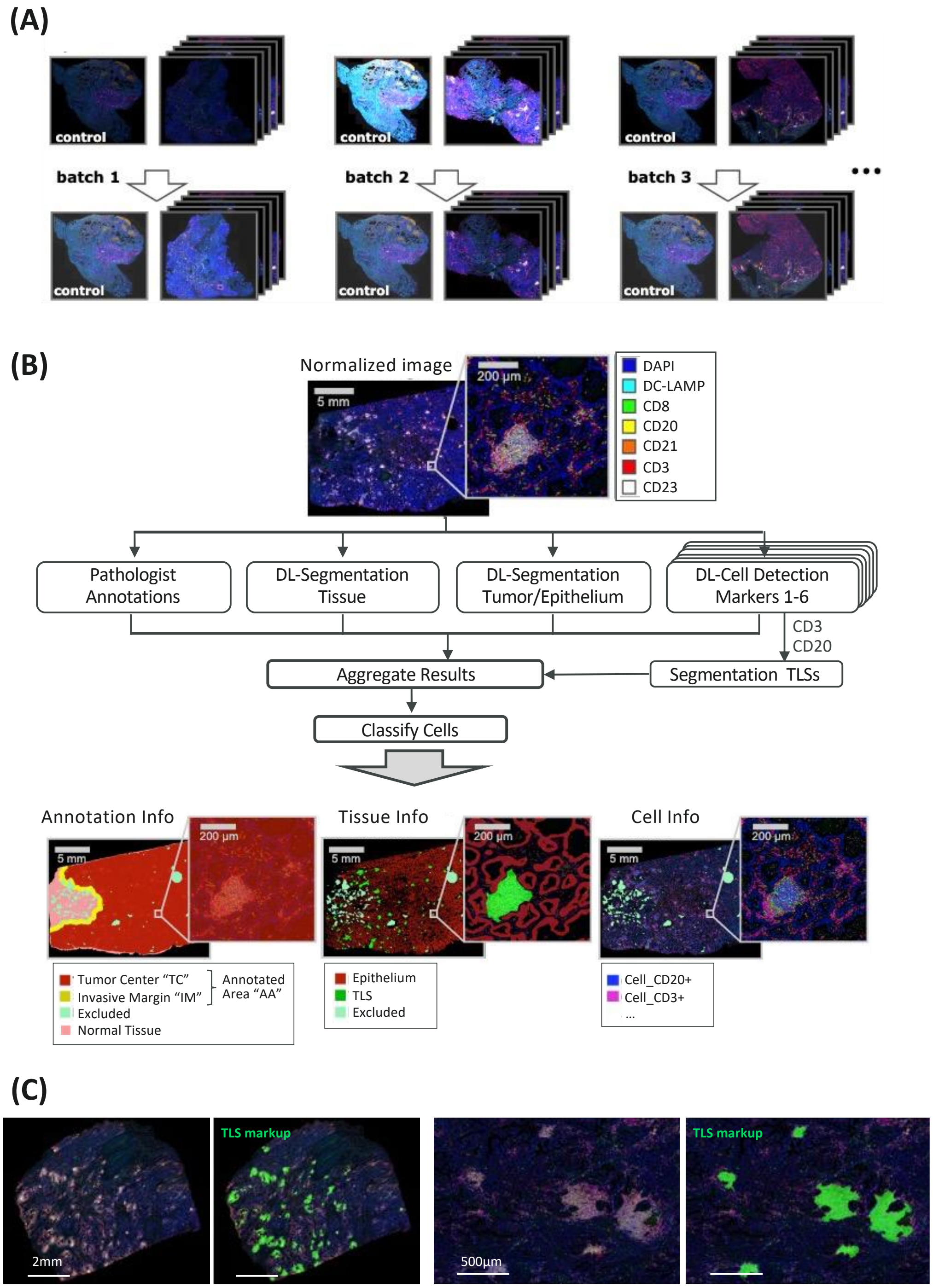

Supplementary Figure 1: Image analysis workflow to specifically detect TLS structures and evaluate their cellular composition. (A) Normalization of the staining intensities using the batch controls images corresponding to serial sections of Tonsil and NSCLC control tissues stained at each experimental run. Slides were imaged using the PhenoImager HT automated imaging system. **(B)** Annotation of the normalized images by pathologists, and development of Deep Learning algorithms to segment the tumor tissues into different tissue areas and detect cell positivity for each marker (CD3, CD8, CD20, CD21, CD23 and DC-LAMP). **(C)** Representative images of TLS / B-cell clusters detection using our image analysis solution. Scale bars are indicated.

SUPPLEMENTAL FIGURE 2

(A)

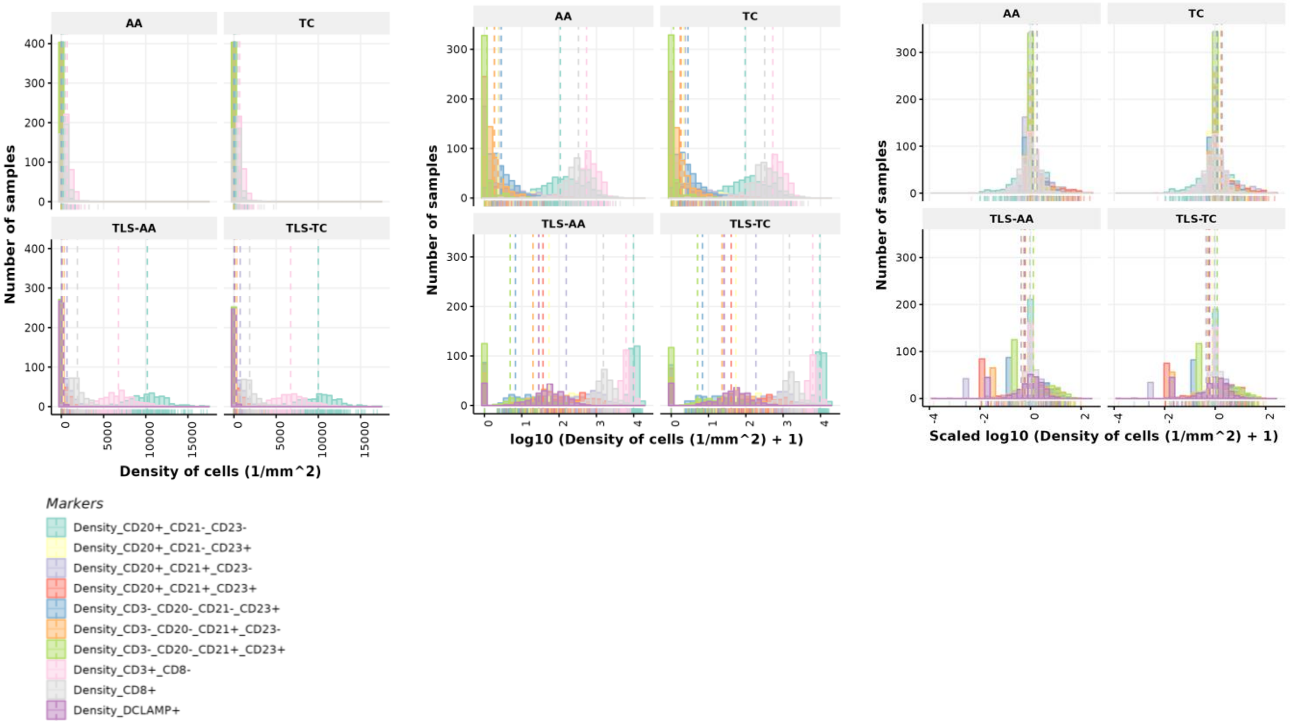

(B)

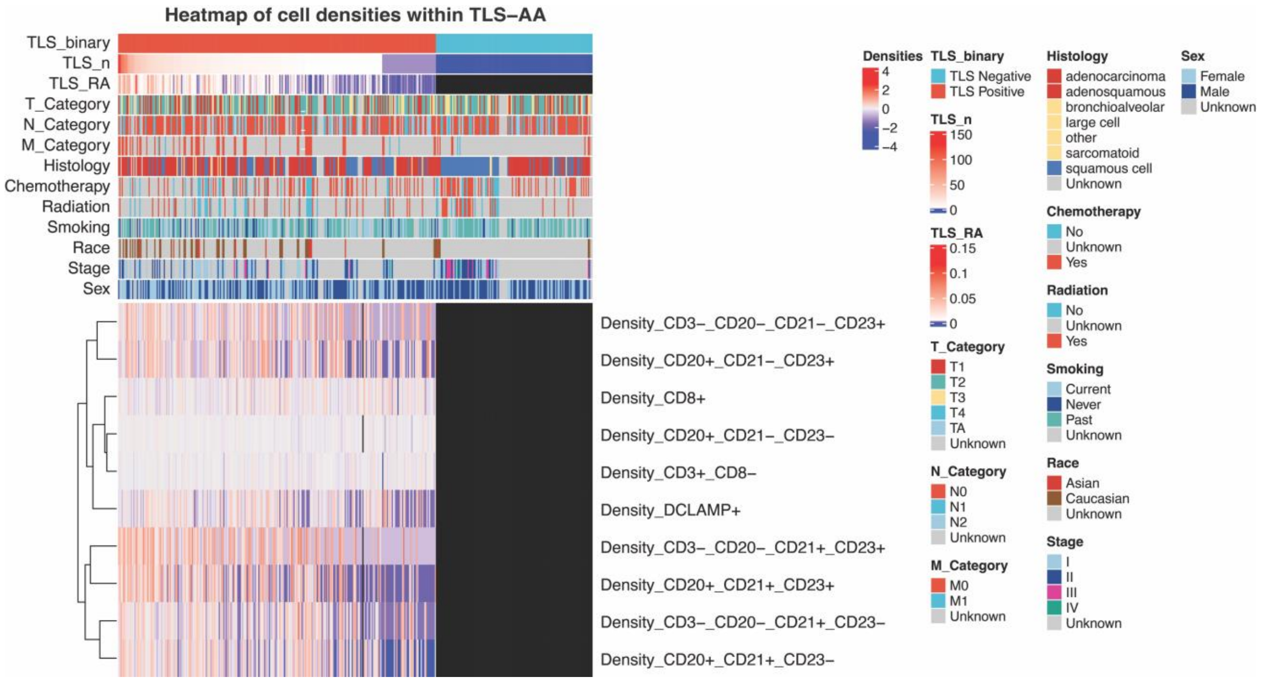

Supplementary figure 2: Cell densities within the different tumor tissue areas and heatmap visualisation using the cell phenotypes densities within TLS structures.

(A) Histograms showing the cell densities in NSCLC samples. The x-axis represents the raw densities (left panel), the densities after logarithmic transformation (base 10) (middle panel) and the scaled densities following the log10 transformation (right panel). The y-axis represents the number of samples. The dotty lines represent the median cell density values of the cell phenotypes. (**B)** Heatmap showing the scaled density (following the $\log_{10}$ transformation) of ten cell phenotypes in the TLS-specific 'TLS-AA' region and their association with sex, stage, race, smoking status, therapy (radiation and chemotherapy), histology and other covariates. Individual patients are represented in each column. Each row represents the cell density of a specific cell phenotype located within the TLS-AA region.

SUPPLEMENTAL TABLE 1

C-index, Hazard ratio, 95% CI and p-values of divers TLS features and cell densities (in log_{10} scaled) within different tissue areas.

Variables	Region	cindex	HR	HR95lo	HR95hi	pval
TLS Relative area	N/A	0.539	0.000	0.000	0.469	0.041
TLS number	N/A	0.559	0.982	0.964	1.001	0.070
TLS Relative area scaled	N/A	0.539	0.766	0.594	0.989	0.041
TLS number scaled	N/A	0.559	0.757	0.561	1.023	0.070
TLS binary TLS Positive	N/A	0.548	0.757	0.565	1.015	0.063
Age	N/A	0.597	1.038	1.021	1.057	0.000
AA (Total annotated area)	N/A	0.525	1.001	0.997	1.005	0.722
Density CD20+CD21-CD23-	AA	0.574	0.691	0.554	0.863	0.001
Density CD20+CD21-CD23-	TC	0.575	0.685	0.551	0.853	0.001
Density CD20+CD21-CD23-	TLS-AA	0.487	0.750	0.160	3.517	0.715
Density CD20+CD21-CD23-	TLS-TC	0.540	1.576	0.358	6.941	0.547
Density CD20+CD21-CD23+	AA	0.567	0.640	0.450	0.911	0.013
Density CD20+CD21-CD23+	TC	0.564	0.629	0.434	0.911	0.014
Density CD20+CD21-CD23+	TLS-AA	0.528	0.958	0.788	1.164	0.663
Density CD20+CD21-CD23+	TLS-TC	0.523	0.959	0.781	1.178	0.690
Density CD20+CD21+CD23-	AA	0.578	0.576	0.404	0.823	0.002
Density CD20+CD21+CD23-	TC	0.570	0.601	0.421	0.859	0.005
Density CD20+CD21+CD23-	TLS-AA	0.573	0.851	0.729	0.992	0.039
Density CD20+CD21+CD23-	TLS-TC	0.569	0.842	0.716	0.990	0.037
Density CD20+CD21+CD23+	AA	0.557	0.717	0.491	1.047	0.085
Density CD20+CD21+CD23+	TC	0.556	0.721	0.483	1.077	0.110
Density CD20+CD21+CD23+	TLS-AA	0.553	0.873	0.745	1.023	0.092
Density CD20+CD21+CD23+	TLS-TC	0.551	0.870	0.739	1.025	0.097
Density CD3-CD20-CD21-CD23+	AA	0.538	0.773	0.494	1.210	0.260
Density CD3-CD20-CD21-CD23+	TC	0.537	0.773	0.493	1.212	0.262
Density CD3-CD20-CD21-CD23+	TLS-AA	0.490	1.037	0.795	1.352	0.789
Density CD3-CD20-CD21-CD23+	TLS-TC	0.507	1.133	0.871	1.472	0.352
Density CD3-CD20-CD21+CD23-	AA	0.545	0.559	0.304	1.026	0.061
Density CD3-CD20-CD21+CD23-	TC	0.549	0.560	0.307	1.020	0.058
Density CD3-CD20-CD21+CD23-	TLS-AA	0.584	0.762	0.617	0.942	0.012
Density CD3-CD20-CD21+CD23-	TLS-TC	0.580	0.793	0.639	0.984	0.035
Density CD3-CD20-CD21+CD23+	AA	0.548	0.293	0.076	1.123	0.073
Density CD3-CD20-CD21+CD23+	TC	0.543	0.354	0.093	1.349	0.128
Density CD3-CD20-CD21+CD23+	TLS-AA	0.564	0.819	0.630	1.066	0.138
Density CD3-CD20-CD21+CD23+	TLS-TC	0.567	0.817	0.625	1.069	0.141
Density CD3+CD8-	AA	0.564	0.552	0.363	0.839	0.005
Density CD3+CD8-	TC	0.567	0.543	0.363	0.813	0.003
Density CD3+CD8-	TLS-AA	0.501	0.626	0.194	2.023	0.434
Density CD3+CD8-	TLS-TC	0.506	0.503	0.142	1.785	0.288
Density CD8+	AA	0.554	0.670	0.441	1.019	0.061
Density CD8+	TC	0.559	0.648	0.437	0.960	0.031
Density CD8+	TLS-AA	0.545	1.470	0.778	2.778	0.235
Density CD8+	TLS-TC	0.486	0.929	0.578	1.493	0.761
Density DCLAMP+	TLS-AA	0.511	0.949	0.745	1.209	0.671
Density DCLAMP+	TLS-TC	0.507	0.938	0.737	1.196	0.607

SUPPLEMENTAL FIGURE 3

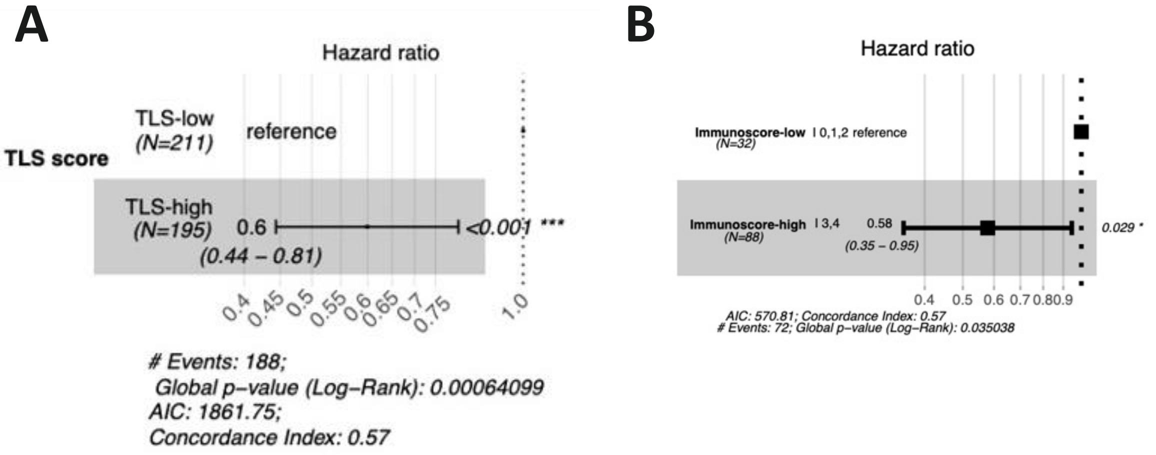

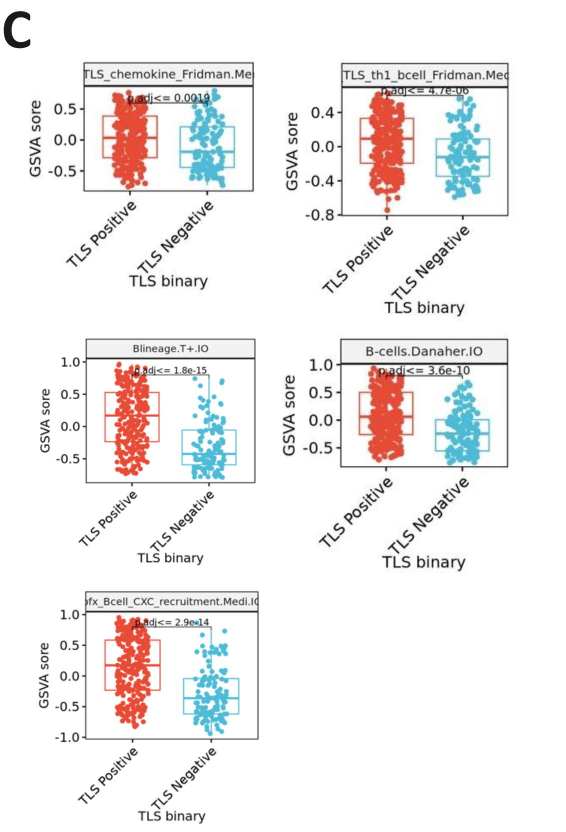

D

Number of samples			
		**TLS status**	
		**Hi**	**Low**
**EGFR status**	**EGFRm**	74	39
	**EGFRwt**	104	144

FDR adjusted P-values from hypergeometric test			
		**TLS status**	
		**Hi**	**Low**
**EGFR status**	**EGFRm**	4.95E-05	1.00E+00
	**EGFRwt**	1.00E+00	4.95E-05

Supplementary figure 3: Overall survival analysis of TLS score and Immunoscore, TLS presence correlation with gene expression-based B cells and TLS signature scores, and TLS score correlation with EGFR mutational status. (A-B) Univariate Cox analysis of TLS-high and TLS-low (reference) groups **(A)** and Immunoscore-high and -low (reference) groups **(B)**. **(C)** Box plot comparing of gene expression-based signature score in TLS-present and TLS-absence samples. **(D)** Number of TLS Score -high and -low cases in EGFR mutant and EGFR wildtype cohorts (top panel) and statistical significance with FDR adjusted p-values from a hypergeometric test (lower panel).

SUPPLEMENTAL FIGURE 4

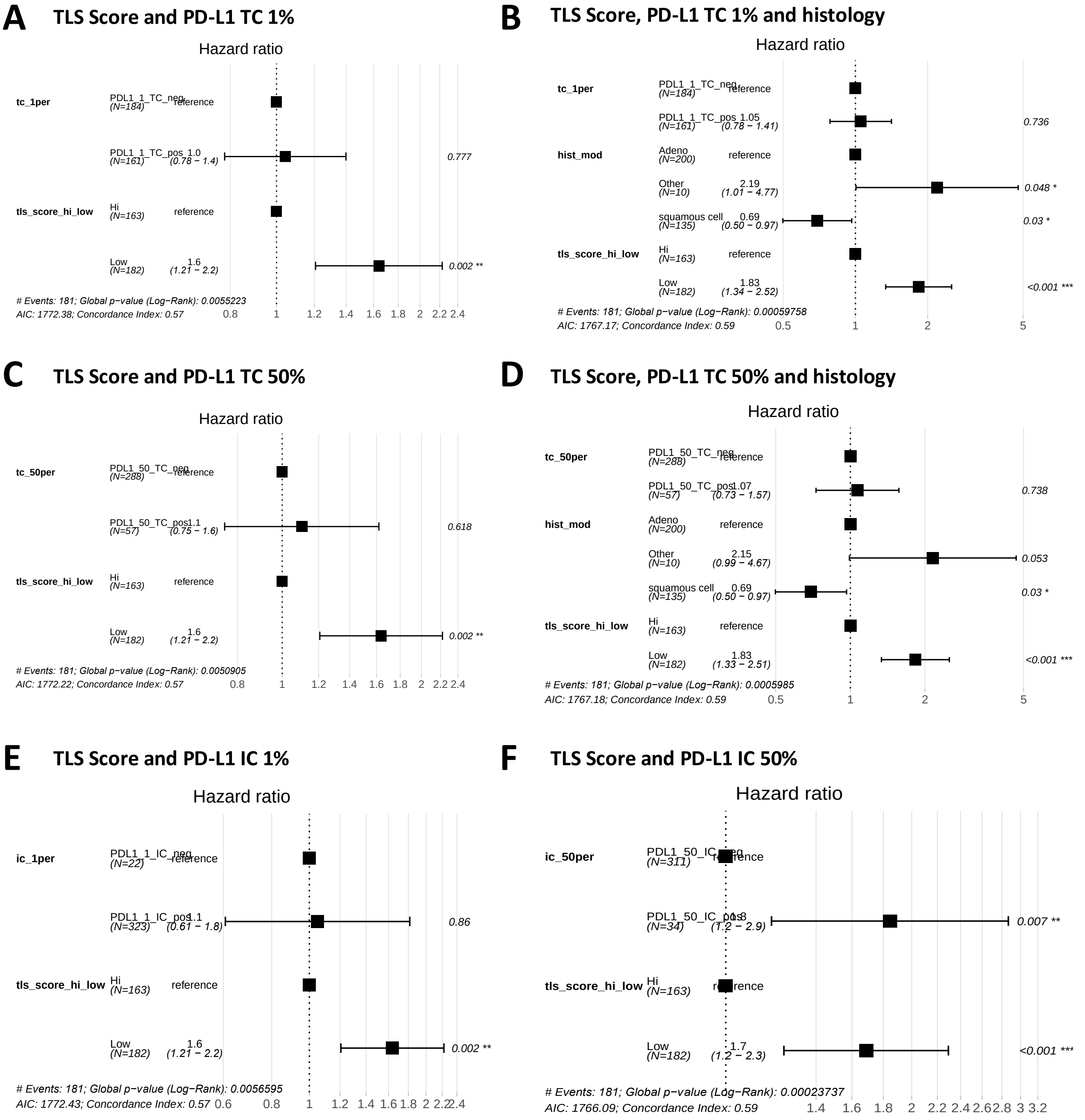

Supplementary figure 4: TLS score is prognostic after adjusting for PD-L1 status in tumor and immune cells. Forest plots showing the hazard ratios and 95% confidence interval of TLS Score adjusted for the effect of PD-L1 in tumor cells (A-D) and immune cells (E-F) using multivariable Cox regression analysis and overall survival data. (A) PD-L1 status in tumour cells (stratified using cutoff 1%), (B) PD-L1 status in tumour cells (1% cutoff) and histology, (C) PD-L1 status in tumour cells (stratified using cutoff 50%), (D) PD-L1 status in tumour cells (50% cutoff) and histology, (E) PD-L1 status in immune cells (1% cutoff) and PD-L1 status in immune cells at 50% cutoff (F).
